# OralImmunoAnalyser: a software tool for immunohistochemical assessment of oral leukoplakia using image segmentation and classification models

**DOI:** 10.3389/frai.2024.1324410

**Published:** 2024-02-26

**Authors:** Zakaria A. Al-Tarawneh, Maite Pena-Cristóbal, Eva Cernadas, José Manuel Suarez-Peñaranda, Manuel Fernández-Delgado, Almoutaz Mbaidin, Mercedes Gallas-Torreira, Pilar Gándara-Vila

**Affiliations:** ^1^Computer Science Department, Mutah University, Karak, Jordan; ^2^Centro Singular de Investigación en Tecnoloxías Intelixentes da USC, Universidade de Santiago de Compostela (USC), Santiago de Compostela, Spain; ^3^Oral Medicine, Oral Surgery and Implantology Unit, MedOralRes Group of University of Santiago, Santiago de Compostela, Spain; ^4^Pathological Anatomy Service, University Hospital Complex of Santiago (CHUS), Santiago de Compostela, Spain; ^5^Department of Forensic Sciences and Pathology, University of Santiago, Santiago de Compostela, Spain

**Keywords:** image processing, immunohistochemical image, machine learning, oral cancer, oral potentially malignant disorders, software

## Abstract

Oral cancer ranks sixteenth amongst types of cancer by number of deaths. Many oral cancers are developed from potentially malignant disorders such as oral leukoplakia, whose most frequent predictor is the presence of epithelial dysplasia. Immunohistochemical staining using cell proliferation biomarkers such as ki67 is a complementary technique to improve the diagnosis and prognosis of oral leukoplakia. The cell counting of these images was traditionally done manually, which is time-consuming and not very reproducible due to intra- and inter-observer variability. The software presently available is not suitable for this task. This article presents the OralImmunoAnalyser software (registered by the University of Santiago de Compostela–USC), which combines automatic image processing with a friendly graphical user interface that allows investigators to oversee and easily correct the automatically recognized cells before quantification. OralImmunoAnalyser is able to count the number of cells in three staining levels and each epithelial layer. Operating in the daily work of the Odontology Faculty, it registered a sensitivity of 64.4% and specificity of 93% for automatic cell detection, with an accuracy of 79.8% for cell classification. Although expert supervision is needed before quantification, OIA reduces the expert analysis time by 56.5% compared to manual counting, avoiding mistakes because the user can check the cells counted. Hence, the SUS questionnaire reported a mean score of 80.9, which means that the system was perceived from good to excellent. OralImmunoAnalyser is accurate, trustworthy, and easy to use in daily practice in biomedical labs. The software, for Windows and Linux, with the images used in this study, can be downloaded from https://citius.usc.es/transferencia/software/oralimmunoanalyser for research purposes upon acceptance.

## 1 Introduction

Oral cancer represents the sixteenth most deadly type of cancer worldwide and represents a serious and growing public health problem (IARC-OMS, 2020). A significant part of oral cancers develop from potentially malignant disorders such as oral leukoplakia (Warnakulasuriya et al., 2020). So, its identification and intervention predictor of malignant transformation in premalignant stages could be key in reducing mortality, morbidity, and the cost of treatment associated with oral cancer (Humayun and Prasad, 2011). One of the main predictors of malignant transformation of oral leukoplakia is the presence of epithelial dysplasia (Warnakulasuriya, 2001; Reibel, 2003; Gandara-Vila et al., 2018). However, this diagnosis is based on a static image and it has been shown that there is great inter- and intra-examiner variability when evaluating the presence or absence of dysplasia, as well as its degree (Warnakulasuriya, 2001; Kujan et al., 2007). Clinical and histopathological analysis by the exclusion of other disorders is the conventional diagnosis of oral leukoplakia (Warnakulasuriya et al., 2007; van der Waal, 2009). Machine learning was used in clinical images to predict the high risk of dysplasia and evolution to cancer (Ferrer-Sánchez et al., 2022).

However, changes at the molecular level occur before this histological evaluation (Mehrotra et al., 2005), so the use of immunohistochemical staining that reveals the expression of the cell proliferation biomarkers, such as ki67, could be a complementary technique to improve diagnosis and prognosis (Reibel, 2003). Some studies show that the expression of ki67 staining could be used to estimate the degree of dysplasia in oral leukoplakia and the risk of malignant transformation in oral potentially malignant disorders.

The cell counting of these immunohistochemical images is classically carried out visually and manually with the help of devices designed for this purpose. These manual counting techniques are very time-consuming, and the experts only count a reduced number of cells (normally 100). Besides, there is a high intra- and inter-observer variability in the results obtained, which hinders its reproducibility (Seidal et al., 2001). These limitations could be alleviated by using image analysis software (Mungle et al., 2017). The development of computerized methods to analyze biopsies in order to make diagnostic and prognostic assessments, mainly based on cell morphology and architecture, is an open challenge (Irshad et al., 2014). The Aperio system^1^ (Morais et al., 2019) is a commercial solution, which has been used in the immunohistochemical study of oral lesions, and offers solutions for slide glass scanning and automatic analysis of immunohistochemical staining. ImageJ (Rueden et al., 2017) is a freely accessible software that has also been used in the immunohistochemical study of oral lesions (Park et al., 2020). It provides utilities to create macros or plugins, but this process requires specialized knowledge of computer language and programming skills. Other works propose automatic algorithms to analyze the image, normally counting cells or other measures (Lu et al., 2018). In general, the available alternatives to analyze the immunohistochemical samples (Paravani et al., 2010) have some of the following drawbacks: (1) they usually have a high cost and low flexibility to face artifacts in the images or differences among samples, and (2) they do not allow the expert supervision before the quantification. In previous works, we developed software tools that combine the automatic processing of the image with a friendly graphical user interface (GUI) to review the recognition process before image quantification in other problems. STERapp (Mbaidin et al., 2021) performs stereological analysis from histological images of fish gonads to estimate their fecundity. CystAnalyser (Cordido et al., 2020) studies histological images of cystic liver and kidney in order to provide their cystic index, number of cysts and cyst profile according to their size. Both pieces of software use sophisticated algorithms of image analysis and machine learning to automatically recognize and classify the objects of interest in the image. When the automatic recognition provided is not suitable for experts, due to the inherent complexity of microscopic images, they provide a friendly GUI, that allows the experts to review the recognition before measuring and counting them.

This paper proposes the software OralImmunoAnalyser, which quantitatively estimates the immunohistochemical expression of the molecular marker ki67 in oral leukoplakia. This software is intended to fulfill the following requirements: (1) provide a friendly GUI to interactively work with the images and draw the region of analysis; (2) use advanced image analysis and machine learning algorithms to automatically detect and classify cells in the images; (3) estimate automatically various statistical measures and counts in the images, calculating the positivity in the basal, medium and superior layers of the epithelium; (4) allow data sharing among researchers and to review the results at any time; and (5) be fast enough to analyze images in real-time.

The paper is organized as follows. Section 2 describes the materials used to obtain the immunohistochemical images. Section 3 describes: (1) the architecture and functionality provided by OralImmunoAnalyser; (2) the image analysis and machine learning algorithms used by the software to detect and classify the cells; and (3) the algorithm to calculate the three epithelial layers. Section 4 discusses the results. Finally, Section 5 summarizes the main conclusions and proposals for future work.

## 2 Materials

The tissue samples analyzed correspond to patients from the Department of Oral Medicine, Oral Surgery and Implantology of the University of Santiago de Compostela, where they were diagnosed, clinically and histologically, with oral leukoplakia. The participants consent to the use of their clinical, histological, and photographic data, treated anonymously, in the present study. This study has the approval of the Santiago-Lugo Research Ethics Committee, with registration code 2020/470.

The histological samples were analyzed at the Pathological Anatomy Service of the Complejo Hospitalario Universitario de Santiago de Compostela. Surgical specimens were fixed in 10% buffered formalin for a maximum of 24 h, and embedded in paraffin. After the study with the usual hematoxylin-eosin techniques, new sections of different degrees of epithelial dysplasia were made for the immunohistochemical study using monoclonal mouse anti-human ki67 antigen (clone MIB-1) (Dako, Denmark), following the manufacturer's recommended instructions. Cells labeled by the antibody show a nuclear staining pattern in a brown color. Once the area with the highest number of stained cells has been selected, a photograph was acquired using an Olympus BX51 microscope connected to an Olympus Camera DP70, using a magnification of 20X but the same can be made with a major magnification. The size of the acquired images is 4, 080 × 3, 072 pixels. This image was archived in TIF format.

## 3 Methods

**Figure 1:**
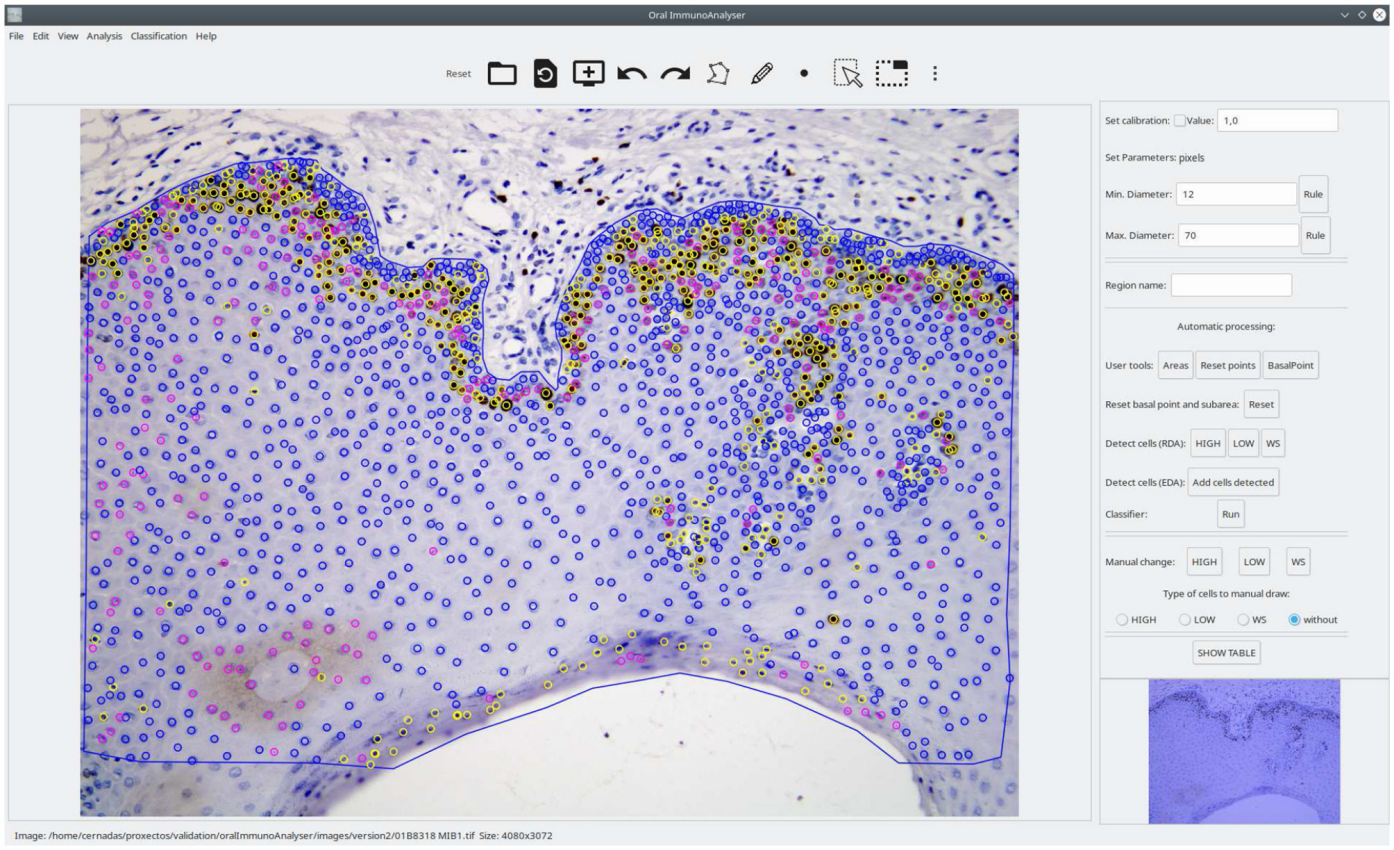
Screenshot of the software OralImmunoAnalyser. In the region of analysis (defined by the blue line), the color of the dots shows the category of the cells: yellow (highly stained), pink (low stained), and blue (no-stained).

OralImmnunoAnalyser (OIA) is a desktop application that runs on a general-purpose computer on the Linux and Windows operating systems. It has been written in the C++ programming language using the GTK+ (GIMP Tool Kit) library^2^ to develop the GUI and the OpenCV library^3^ to develop the automatic algorithms to process the images. Figure 1 shows the GUI of OIA with a typical immunohistochemical image loaded, processed, and reviewed by the expert, and with the lateral panel displayed.

### 3.1 System architecture

The architecture of OralImmunoAnalyser is modular and extensible, being composed of the classical three layers: (1) the GUI layer interacts with the user with editing tools, including modules to draw and manage objects, set preferences or interact with the software; (2) the logic application layer contains modules to detect the cells, classify them, train the classifier and calculate the statistical results; and (3) the persistent layer to store all the data needed and calculated by the software, including modules to save the overlays on the image and the statistical results. The overlays on the image, which contain the analysis supervised by the experts, are stored in the popular text format XML (Extensible Markup Language). The statistical results, calculated from the overlays, are stored in the known text format CSV (Comma-Separated Values), which is portable, and can be imported from other spreadsheet software for further use.

### 3.2 Funcionality of OralImmunoAnalyser

**Figure 2:**
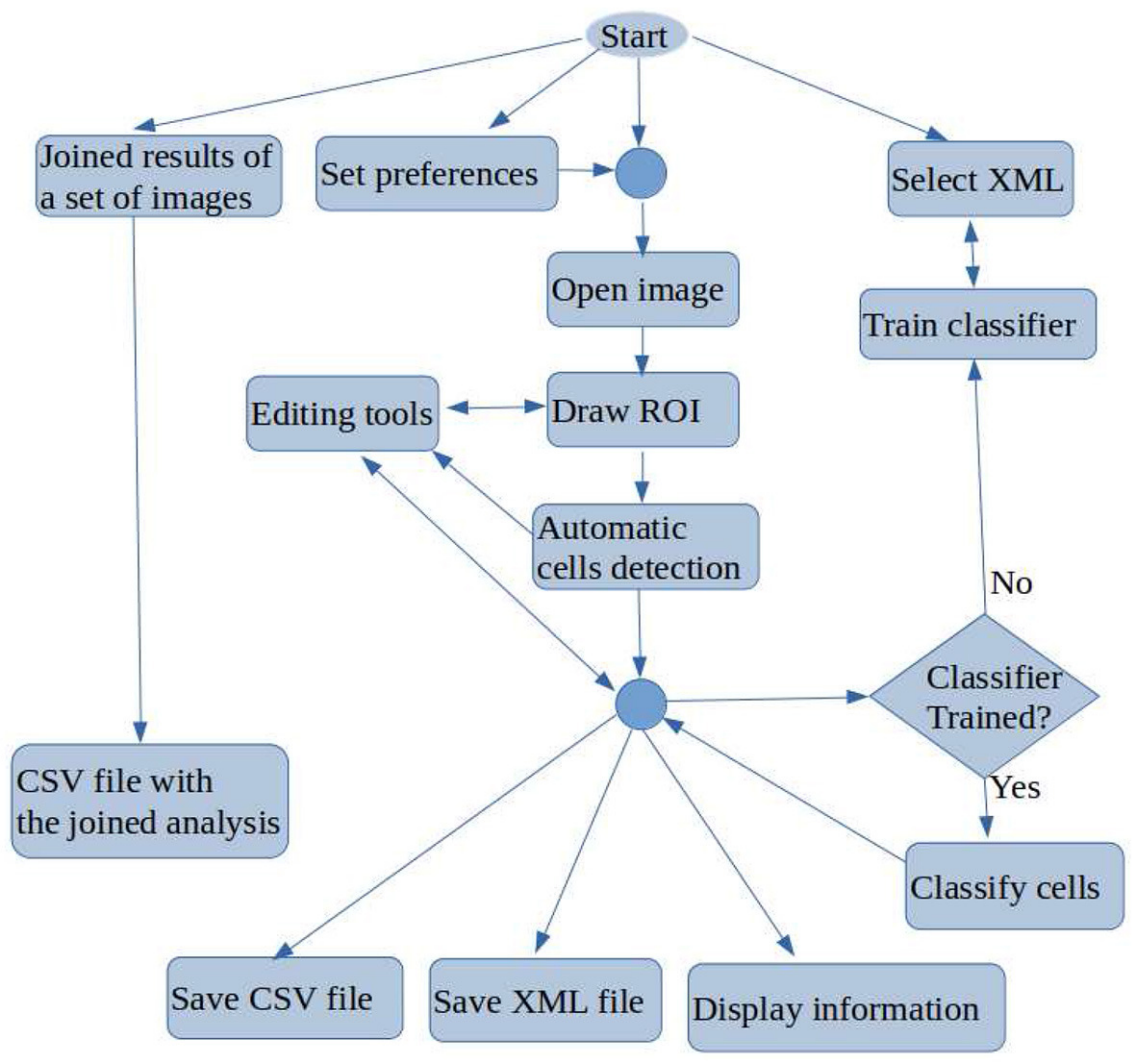
Flowchart containing the main tasks of OralImmunoAnalyser.

Figure 2 shows a flowchart with the main functionality of OIA, which is accessible from its GUI. A typical working session for a user should have the following actions: (1) open a microscopic image; (2) draw manually the region of interest (ROI) for the analysis using the editing tools; (3) detect automatically the cells into the ROI; (4) classify automatically the cells detected; (5) go to expert's supervision, as described below; (6) save the overlays drawn on the image into XML files; (7) export the statistical measures and counts to CSV files; and (8) every time the user can do the following optional functionality: set preferences, set calibration and diameters, save joined results of a set of images, and train the classifier.

Once the image is loaded, a freehand region must manually be drawn using the edition tools, in which the user wants to do the count. After drawing and selecting the ROI, the buttons of the lateral panel can be used to automatically detect the cells in the ROI (the two algorithms included are described below). Next, the classifier can be run in order to label each detected cell as “highly stained,” “low stained,” and “no-stained.” This automatic analysis may be not perfect due to the complexity of this type of image, so the expert supervises the detection and classification results using the editing tools in the following ways: (1) deleting a set of detected cells; (2) changing the category of the detected cells; and (3) adding new cells specifying their category labels.

Once the detection and classification of cells are reviewed, the expert must mark with the mouse a point on the image indicating to the software where is the basal part of the ROI in the study. This step is necessary to do the count for the basal, medial, and superior areas of the ROI, otherwise, the count is only done globally. The overlays of the analysis must be saved into the XML file in order to review or export joined results in a future time. Given the basal point and the ROI under study, some geometrical computations can be done to divide the ROI into three areas: basal, medial, and superior. The areas calculated can be visualized by clicking the button *Areas* in the lateral panel (see Section 3.5 for a detailed description).

The working preferences of OIA can be set going to the menu *File* → *Set preferences*, in which you can set: (1) the working directories for images, overlays, and results; (2) the width of points and lines; (3) the color of the overlays for each cell category; and (4) the color of the basal point. The user preferences can be saved permanently for the next sessions. OralImmunoAnalyser allows setting the spatial calibration, which is the relation between pixels in the image and real values (microns), or working in pixels units. If the calibration is set, the results are provided in real measures instead of pixels. The user must provide OIA the minimum and maximum diameter of the cells to detect for an optimal operation of the automatic algorithms, which can be set by writing in the *Preferences* dialog or graphically by drawing a straight line with the editing tools of the lateral panel. The preferences can be stored for future working sessions. OralImmunoAnalyser allows to export joined results of a set of images going to the menu *Analysis* → *XML Files*, which opens a dialog screen to select the XML files and the output CSV file. To do this task, the images had to be analyzed, supervised by the expert, and saved as overlays in XML files (one per image). OralImmunoAnalyser also allows training the classifier by going to the menu *Analysis* → *Train classifier*. More details of this functionality can be read in the user guide, provided as Supplementary material.

### 3.3 Image analysis algorithms for cell detection

To detect the cells in the image, it is split into pixels belonging to objects that must be detected (in our case cells) and pixels belonging to the background. This process is a simplification of the image representation called segmentation, a very hot topic in computer vision (Sonka et al., 2007). Since the area of cells is not relevant to our objectives, the segmented regions are managed as points by the software. The segmentation process employs the following properties of the image: (1) pixels of an object are similar with respect to some property in the image, such as color, grey level, texture, etc.; and (2) adjacent regions, e.g., object and background, are significantly different for some image property. The first paradigm developed the region detection algorithms (RDA) and the second the edge detection algorithms (EDA). Both approaches are closely related because the boundaries of a region are the edges surrounding the region.

**Figure 3:**
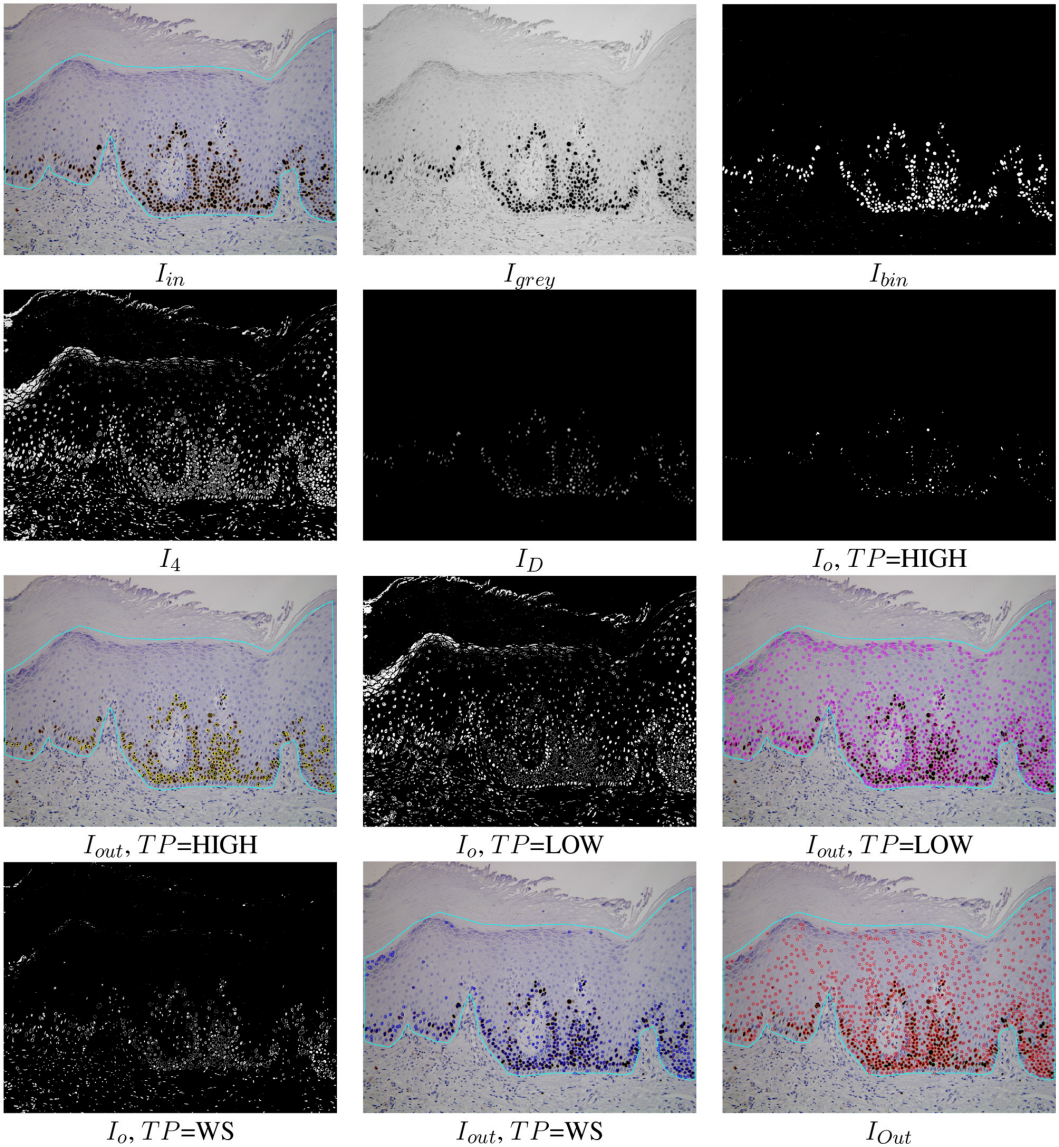
Examples of the automatic processing of immunohistochemical images using the proposed RDA for different types of processing (see Algorithm 1 for the meaning of *I*_*in*_, *I*_*grey*_, *I*_*bin*_, *I*_4_, and *I*_*O*_). The *I*_*out*_ images show the cells detected overlapped to the original images for each option *TP* and the cell detection of all options merged (lower right image *I*_*Out*_).

Our RDA approach is a combination of image processing techniques that encloses the following stages: (1) detect the highly stained regions in the image; (2) split highly stained regions when some cells are joined; and (3) detect low-stained and no-stained cells. We use the color space Lab, also known as CIELAB, to process the image due to: (a) its better perceptual linearity, compared to the color space RGB of image acquisition; and (b) its robustness to illuminance variances (Cernadas et al., 2017). Let *I*_*in*_ be the RGB immunohistochemical image, *d*_*min*_ and *d*_*max*_ the minimum and maximum diameters of the cells to be detected, and *TP* the user option. The value of *TP* can be set in the lateral panel by clicking the **HIGH**, **LOW** or **WS** button after the label *Detect cells (RDA)*. Algorithm 1 summarizes the proposed RDA and Figure 3 shows visual examples of intermediate stages in the image processing.

**Algorithm 1:**
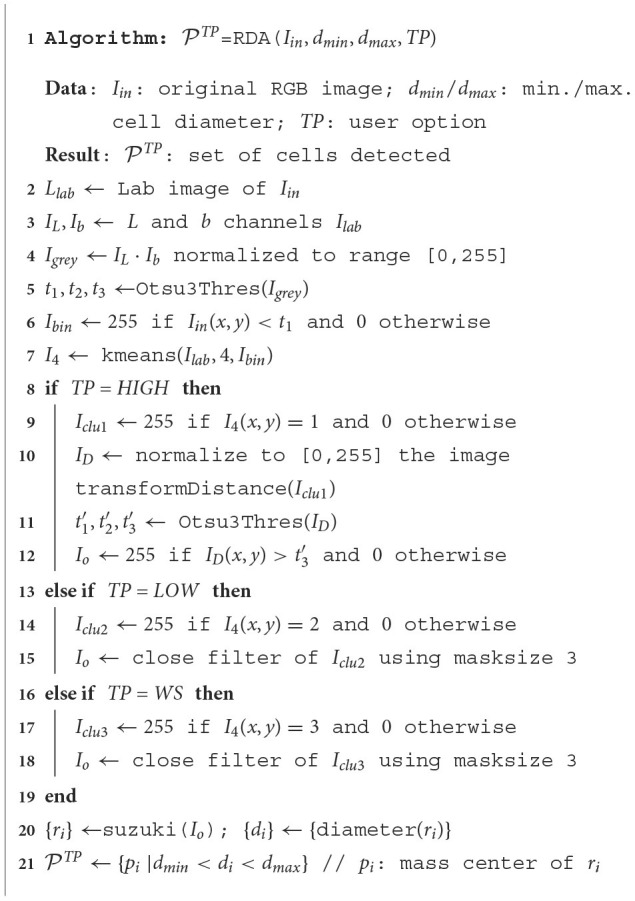
RDA to detect cells in immunohistochemical images.

As mentioned, the original RGB image *I*_*in*_ is converted to the Lab color space giving the image *I*_*lab*_. Then the channels *L* and *b* of *I*_*lab*_ are multiplied and the product is normalized to the range [0,255], giving the image *I*_*grey*_. The *L* channel of the Lab image represents the image intensity and the *b* channel the image yellowness. The multiplication of both channels gives an image with the lowest levels in the more stained regions (*I*_*grey*_ in Figure 3). Thresholding is applied on *I*_*grey*_ in order to create the binary image *I*_*bin*_. The selection of the optimal threshold is critical, and it is commonly selected by trial and error in much of the available software, such as ImageJ. We select this value from the statistical properties of each image using the multi-level Otsu's method (Otsu, 1979), implemented by the Otsu3Thres function in Algorithm 1. The optimal threshold considered was the lowest value *t*_1_ of the three calculated by Otsu3Thres(*I*_*grey*_). The binary image *I*_*bin*_ (see Figure 3), containing white in the more stained pixels and black in the remaining pixels, is calculated thresholding the image *I*_*grey*_ using *t*_1_ (in this case, *t*_1_ = 69) and taking the values less than *t*_1_ as white. Black and white pixels in the *I*_*bin*_ image represent background and highly stained cells, respectively.

Afterward, *k*-means clustering (Duda et al., 2000), implemented by the kmeans function in Algorithm 1, is used to group the pixels in the color image *I*_*lab*_ according to color similarity into four clusters. The *I*_*bin*_ image is used as a seed for the cluster prototypes, representing the two extremes that must be discriminated. The application of *k*-means returns the image *I*_4_ whose pixels are labeled by their cluster (four labels in our case), shown in Figure 3. The 0-th cluster represents the background and includes pixels in *I*_*lab*_ with a color similar to the pixels in *I*_*lab*_ that are black in *I*_*bin*_. Analogously, the 1st cluster represents the highly stained regions and contains pixels in *I*_*lab*_ with color similar to the pixels in *I*_*lab*_ that are white in *I*_*bin*_. Finally, the 2nd and 3rd clusters may represent low or non-stained regions, including pixels with intermediate color values in *I*_*lab*_ that are less similar to the seed pixels (both black and white). The binary images *I*_*clu*1_ (highly stained regions) and *I*_*clu*2_, *I*_*clu*3_ (low and non-stained regions), are created with values 1 in the pixels of the corresponding cluster and 0 in the remaining pixels. Each cluster is associated with a different button in the GUI and to a value of the user option *TP* in RDA: *I*_*clu*1_ with *TP*=HIGH, *I*_*clu*2_ with *TP*=LOW and *I*_*clu*3_ with *TP*=WS. These binary images are the *I*_*O*_ images in Figure 3 for *TP*=LOW and *TP*=WS options, after applying a close morphological filter with mask size three to remove small holes.

Since the highly stained cells are frequently joined, we apply the distance transform algorithm (function transformDistance in Algorithm 1) to the image *I*_*clu*1_, which provides the derived representation of the binary image, where the value of each pixel is replaced by its distance to the nearest background pixel. This resulting image *I*_*D*_ (shown in Figure 3) is normalized to the range [0,255] and thresholded using the highest value provided by Otsu3Thres(*I*_*D*_) $t_{3}^{\prime }$ (in this example $t_{3}^{\prime }$=101). The *I*_*o*_ image for *TP*=HIGH is 255 if $I_{D}\left(x,y\right)>t_{3}^{\prime }$ and 0 otherwise (see Figure 3). The contours of the cells, or external regions, are extracted from the thresholded image *I*_*o*_ (see Figure 3) using the algorithm proposed in Suzuki and Be (1985), implemented by function suzuki in Algorithm 1. A detected contour *r*_*i*_ is considered a true cell if its diameter *d*_*i*_ is between the minimum and the maximum diameter specified by the user, i.e., *d*_*min*_ < *d*_*i*_ < *d*_*max*_. In this case, the detected cell is the centroid (mass center) of the region. Finally, the sets $P^{HIGH}$, $P^{LOW}$ and $P^{WS}$ for *TP*=HIGH, *TP*=LOW and *TP*=WS, respectively, are the sets of cells (represented as points) detected applying the Suzuki algorithm that verify *d*_*min*_ < *d*_*i*_ < *d*_*max*_.

The EDA approach included in the software is the multi-scale Canny filter, proposed by Mbaidin et al. (2023), used also in the Govocitos and STERapp software (Pintor et al., 2016; Mbaidin et al., 2021) to recognize oocytes in histological images of fish gonads. In the current work, we use only a Canny filter tuned with a Gaussian smoothing width σ = 4. The thresholds of the hysteresis process are automatically calculated from the statistical image characteristics using rates of 0.3 and 0.7 for the lower and higher thresholds, respectively.

The RDA approach is accessible from the lateral panel of the GUI in the label *Detect cells (RDA)*, which encloses the three toggle buttons **HIGH**, **LOW** and **WS** to show the point sets $P^{HIGH}$, $P^{LOW}$ and $P^{WS}$, respectively. The EDA approach is also accessible from the label *Detect cells (EDA)* of the lateral panel. The visualization of the detected cells is accumulated, applying an overlapping test to remove cells detected by different approaches. Two detected points are considered as two different cells if their distance is superior to the minimum diameter provided by the user, i.e., the final set P of detected cells is $P\leftarrow \{p_{i}\in P^{TP},p_{j}\in P^{TP},TP\in \{HIGH,LOW,WS,EDA\}|$ distance(*p*_*i*_, *p*_*j*_) > *d*_*min*_}. A set of cells can be added or removed by clicking the previous toggle buttons. Finally, only the detected cells inside the region of analysis provided by the user are visualized in the software. The *I*_*out*_ image in Figure 3 shows the set of cells P overlapped to the original image inside the region of analysis drawn by the user. In this example, 96.25% of cells were correctly detected. The color of points means the cell staining level provided by the classifier (**Run** button after the label *Classifier*).

### 3.4 Machine learning model for cell classification

Once the cells are detected on the image, a supervised machine learning model is used to predict their categories, defined by their stain levels: highly stained cell, low stained cell, and cell without staining. In order to perform this prediction, the model must be trained, i.e., it must learn from a collection of examples (cells) to predict their categories. Each cell is represented by a set of numeric characteristics calculated in the following way: (1) a square region centered in the cell, of size the minimum diameter, is extracted from the original *I*_*lab*_ image; and (2) the average value of the three channels L, a and b of the *I*_*lab*_ image over this region is calculated. In the training, the model learns to predict the cell category using these three average values over the cell square for all the cells in the collection. The model used by OralImmnunoAnalyser to predict the cell category is the support vector machine (SVM) with radial basis function (RBF) kernel because it is one of the best-performing machine learning models for classification (Fernández-Delgado et al., 2014). Specifically, OIA uses the LibSVM implementation (Chang and Lin, 2011) of SVM, accessed through its C++ binding. Although the first version included a pre-trained SVM, the current version allows SVM training. The module *Training panel*, through the submenu *Classification* → *Train classifier* allows the user to set the XML files (generated previously by OIA) that will be used to train the SVM classifier. These files must contain the cells recognized for a collection of images, alongside their category. The collection of images should be representative enough for the classification problem and must contain cells of all the categories. For the training, a maximum number of 1,000 cells is randomly selected from the XML files provided by the user, with similar numbers of cells for each category whenever possible (a minimum number of 10 cells is required for a category to be included in the training). OralImmunoAnalyser performs the tuning of the two hyper-parameters of the SVM (regularization λ and RBF kernel spread σ) using the grid-search method. The performance is evaluated by the Cohen kappa statistic (Carletta, 1996), which measures the agreement between the true and predicted category excluding the agreement by chance. kappa (in %) is defined by Equation 1:


(1) $$\begin{array}{r} \text{kappa}=100\frac{p_{a}-p_{e}}{s-p_{e}},\;p_{a}=\overset{C}{\underset{i=1}{\sum }}N_{ii},\;s=\overset{C}{\underset{i=1}{\sum }}\overset{C}{\underset{j=1}{\sum }}N_{ij}\\ p_{e}=\frac{1}{N^{2}}\overset{C}{\underset{i=1}{\sum }}\left(\overset{C}{\underset{j=1}{\sum }}N_{ij}\right)\left(\overset{C}{\underset{j=1}{\sum }}N_{ji}\right) \end{array}$$

where *N*_*ij*_ is the number of cells of category *i* and that are assigned by the SVM to category *j*, *C* = 3 is the number of categories and *N* is the number of cells. The values of λ and σ used are: $\lambda ={\left\{2^{2i-7}\right\}}_{i=1}^{10}$ and $\sigma ={\left\{2^{-\left(i+1\right)/2}\right\}}_{i=-15}^{0}$. For each combination of hyper-parameter values, the SVM is trained using the *K*-fold cross-validation methodology with *K* = 4, so that *K*−1 = 3 folds are used to train the SVM, and the remaining fold is used to calculate the kappa of the trained SVM. The training and prediction are performed *K* times, rotating the folds each time (i.e., in the first trial folds 1–3 are used for training and fold 4 for testing; the second trial uses folds 2–4 to train, and fold 1 to test; and so on) and averaging kappa over the *K*-test folds. The process is repeated for all the combinations of hyper-parameter values, and the one that achieves the highest average kappa is selected. Finally, the SVM is trained over the whole collection of cells, using the selected combination of hyper-parameter values, and then it is ready to predict the category for new cells.

### 3.5 Calculation of epithelial strata regions

**Figure 4:**
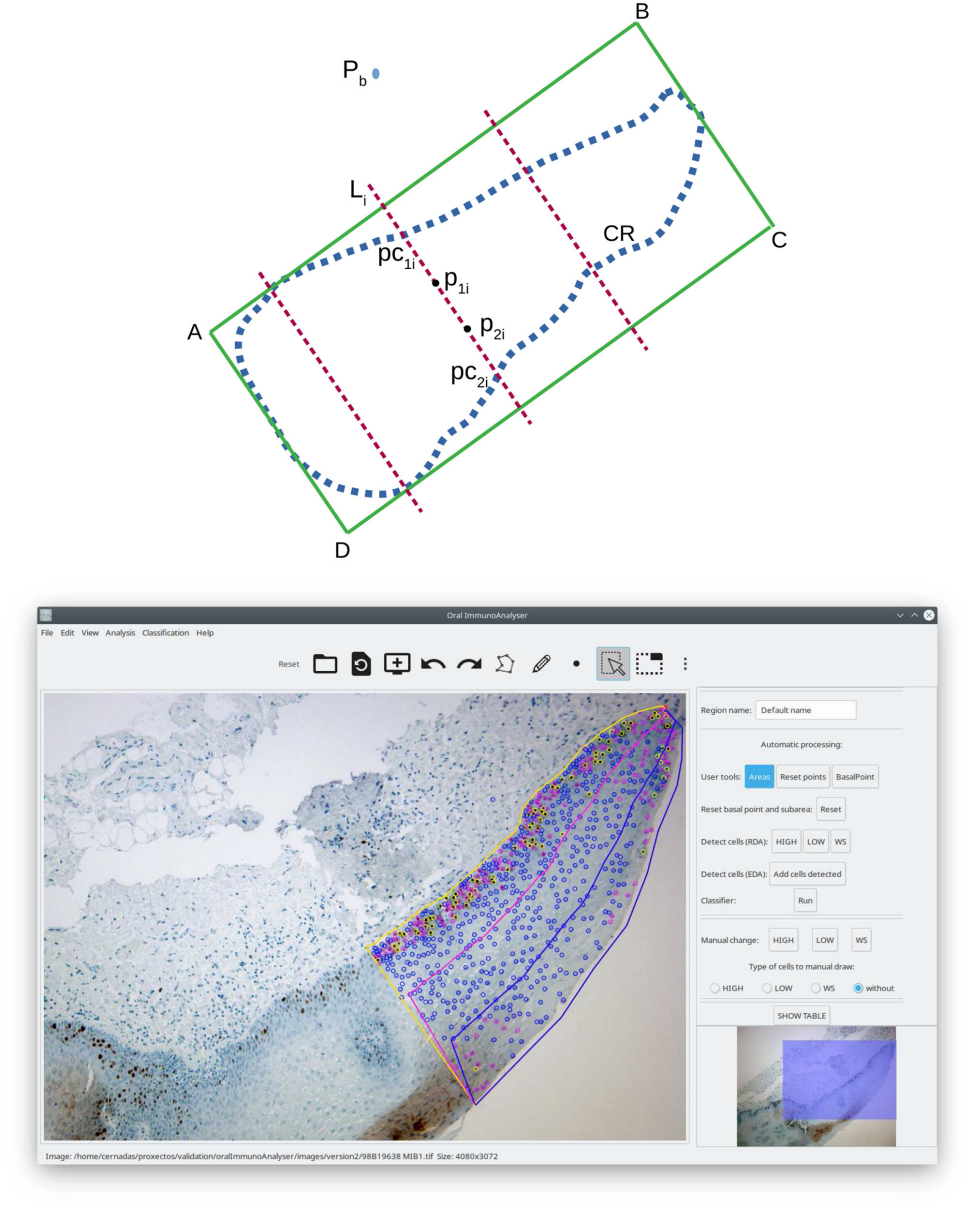
Calculation of the epithelial strata regions: scheme **(upper)** and screenshot of OIA with an example **(lower)**.

Once the image is analyzed (drawing the region of analysis, detecting and classifying the cells in that region), we want to count the cells for each staining level in each epithelial strata. OralImmnunoAnalyser considers three layers: basal, medium, and superior. Let *CR* be the set of points (*x*_*i*_, *y*_*i*_) that define the region of analysis drawn by the user, and let *P*_*b*_ = (*x*_*p*_, *y*_*p*_) be a point marked by the user to indicate the side of the region *CR* where the basal layer is located (outside *CR*). The algorithm to calculate the layer regions, illustrated in the upper panel of Figure 4, includes the following steps: (1) calculate the minimum enclosing rectangle *R* of the region *CR* defined by the points A, B, C, and D; (2) calculate the distance between *P*_*b*_ to each segment of the rectangle (line segments *AB*, *BC*, *CD* and *DA*) in order to determine the closest segment to *P*_*b*_; (3) let, for example, *AB* be the closest segment, then we take the sides of rectangle that are perpendicular and adjacent to *AB*, in our example are *BC* and *DA*; (4) divide the line segments *BC* and *DA* into three equal parts, corresponding to the basal, medial and superior layers; (5) create the list of adjacent rectangle vertices, in our example (A,B,C,D), and calculate the four points of the contour *CR* that are closest to these four vertices, saving the indices of these points in the contour *CR*; (6) create the subregions basal *R*_*b*_, media *R*_*m*_ and superior *R*_*s*_ and copy the contour points *CR* to the subregions; and (7) generate the boundary points among subregions taking a set of *N*_*L*_ lines *L*_*i*_, with *i* = 1, …, *N*_*L*_, parallel to the line segment *BC* between the line segments *AB* and *CD*. For each line *L*_*i*_, calculate its two cross points, *pc*_1*i*_ and *pc*_2*i*_ in the upper panel of Figure 4, with contour *CR*. Then, calculate the two points, *p*_1*i*_ and *p*_2*i*_, of *L*_*i*_ that divide the segment between *pc*_1*i*_ and *pc*_2*i*_ in three pieces of equal length. The points *p*_1*i*_ and *p*_2*i*_ are added to the contours of the subregions basal *R*_*b*_ (only *p*_1*i*_), media *R*_*m*_ (between *p*_1*i*_ and *p*_*i*2_) and superior *R*_*s*_ (only *p*_*i*2_). The right panel of Figure 4 shows overlapped an example of the three epithelial layers calculated.

## 4 Results and discussion

OralImmunoAnalyser was installed in the Odontology Faculty of the University of Santiago de Compostela (Spain) in February 2019 in order to evaluate the software operating in a real environment. The first version included only the edge detection algorithm (EDA) to detect the cells and a pre-trained SVM classifier using a reduced number of cells annotated by the experts. The current (second) version includes both cell detection approaches (EDA and RDA) and it allows the classifier to be trained by the user at any time using the GUI. The experts use OIA in their daily work to do the analysis of the images, for which they were required to detect and classify the cells in the images. Their operations using OIA were logged into XML files for later statistical evaluation of the automatic image analysis and machine learning algorithms incorporated in the software. First, we describe the statistical measures to evaluate their performance and summarize the results achieved from different points of view: automatic detection and classification of cells and performance analysis of the global system.

### 4.1 Statistical analysis

To evaluate the cell detection algorithms, we define a true positive (TP) hit when a cell is correctly detected and a false positive (FP) whenever the user manually deletes the cell using the GUI. A cell is considered a false negative (FN) if the user manually adds it. Once the TP, FP, and FN values are counted for an image, the sensitivity (Se), specificity (Sp), and average precision (AP), in %, are calculated as:


(2) $$\begin{array}{r} Se=100\frac{TP}{FN+TP},\;Sp=100\frac{TP}{FP+TP}\\ AP=100\frac{TP}{TP+FP+FN} \end{array}$$

The performance of the SVM model in the prediction of the cell category *C*∈{highly stained, low stained, and without stain} is evaluated using the Cohen kappa, defined in Equation (2) above, and the accuracy (in %), whose value is 100 multiplied by the number of cells correctly classified by the classifier and divided by the total number of cells. The sensitivity and specificity of each category are also calculated considering that: (1) the TP is the number of cells of category *C*_*i*_ correctly classified by the SVM into the category *C*_*i*_; (2) the FP is the number of cells classified into category *C*_*i*_, but whose true category label is other; and (3) the FN is the number of cells of true category *C*_*i*_ that the classified assigned to other categories.

### 4.2 Detection and classification of cells

The first version of OIA was used to analyze 15 images distributed into four cases of leukoplakia without dysplasia, three of mild dysplasia, two of severe, three carcinomas *in situ*, two infiltrating carcinomas, and one verrucous carcinoma, based on the latest classification recommended by the World Health Organization (El-Naggar et al., 2017). The current version was used to analyze 26 images distributed into 24 that did not present epithelial dysplasia, one case of mild dysplasia, and one case of moderate dysplasia. In both versions, our purpose was to test the software with a wide variability among the immunohistochemical images, due to the differences in epithelial thickness depending on the oral anatomical location, the sample collection, processing and storage, the presence of epithelial dysplasia in different degrees, etc.

**Figure 5:**
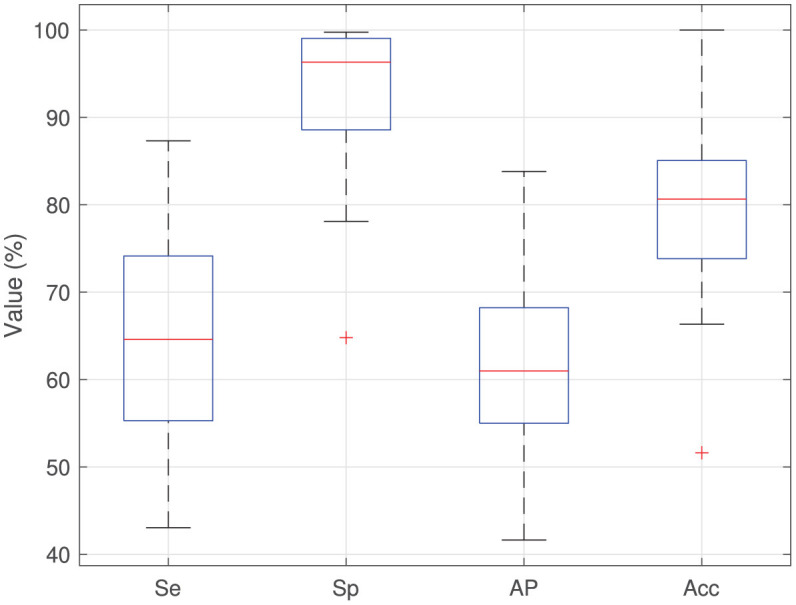
Boxplots of sensitivity (Se), specificity (Sp), average precision (AP), and classification accuracy (Acc) for the images analyzed using the current version of OIA.

Table 1 shows the results achieved for the two versions of OIA, obtained from the analysis of the XML files. The average number of cells in each image was ~1,000. The inclusion of the region-based approach (RDA algorithm) increases the average precision from 41.2% of the first version up to 60.7% to the current version and also increases sensitivity from 41.5% up to 64.4%. That means that, on average, 60.7% of cells in the image are correctly detected and the remaining 39.3% of cells had to be supervised by an expert (adding or deleting cells). Thus, the specificity is much higher than the sensitivity, which means that the experts needed to add cells and delete a small number of cells. The classification accuracy remains more or less constant for both versions, achieving a value of 79.8% with the current version and a kappa value of 60.23%. In order to analyze the influence of the high variability among the immunohistochemical images on the system performance, Figure 5 shows the boxplots of sensitivity, specificity, and average precision for the detection of cells, and the accuracy of the cell classification. The boxes enclose the data between the 25th and 75th percentiles, the red line is the median and the black whiskers extend the extreme data points. The sensitivity box is the largest one (about 20 points), so it is more affected by the image variability than the other measures, existing important differences among images in the proportion of cells that are not detected and must be added by the users. The specificity box is much smaller, so it varies less among images, and its median is much higher. The average precision box is also small, but its median is even lower than sensitivity. Finally, the accuracy box is also very small, so it is not very sensitive to the image variability.

**Table 1:** Sensitivity (Se), specificity (Sp), and average precision (AP) in % of the two versions of OralImmunoAnalyser working in the lab to detect the cells.

**Version**	**#Images**	**#Cells**	**Se**	**Sp**	**AP**	**Acc**.	**Kappa**
First	15	805.3	41.55	99.89	41.16	77.0	–
Current	26	1206.0	64.38	92.98	60.69	79.8	60.23

Average accuracy (Acc) and kappa for cell classification into three categories (highly stained, low stained, and without stain).

In relation to the cell classification, the current version of OIA classifies correctly the 79.8% of the cells with a kappa of 60.23%. More detailed views are in Table 2, which shows the confusion matrix for the category prediction (the value in row *i* and column *j* is 100*N*_*ij*_/*N*, where *N*_*ij*_ and *N* are the same as in Equation 2 above). The diagonal numbers (in bold) give the percentage of cells correctly classified for each category, and the sum of the diagonal gives the classification accuracy. The best performance is provided for cells without staining achieving a high sensitivity (96.9%) and specificity (80.7%) and the worst results are for the low stained cells (sensitivity 42.6% and specificity 62.9%), because the system confuses cells with low and no staining. The reason is that the background staining, which is produced by a defect in the processing of the sample, is very similar to low-intensity brown, and the experts label the cells on these parts as “without stain”. During training, the classifier learns to predict “without stain” for cells with this brown color, and therefore it wrongly classifies cells that are low stained as “without stain”. For cells highly stained, the system has a high specificity (94%) with moderate sensitivity (67.1%), i.e., some highly stained cells are classified as low or without staining, due mainly to artifacts, but very few low stained or without stain cells are classified as “highly stained” (0.7 and 0.08%, respectively).

**Table 2:** Confusion matrix (in %) for cell category prediction (highly stained, low stained, and without stain) for the current version of OralInmunoAnalyser.

		**Predicted category**				
		**Highly**	**Low**	**Without**	**Se**	**Sp**
True category	Highly	**12.14**	3.60	2.36	67.07	93.96
	Low	0.70	**9.15**	11.64	42.54	62.69
	Without	0.08	1.80	**58.52**	96.89	80.69

The columns Se (Sensitivity) and Sp (Specificity) provide the system sensitivity and specificity for each class respectively. The diagonal values that are the percentages of cells of each category correctly classified in bold.

The assessment of ki67 in breast cancer (Nielsen et al., 2020) recommends labeling the cells as positive and negative. If, in our system, highly stained cells were considered positive and low stained and without stained cells were considered negative, the results in Table 2 would achieve an accuracy of 93.6% and a kappa of 75.66%, maintaining the sensitivity and specificity for the positive cells and with a sensitivity and specificity of 98.92 and 93.66% respectively for the negative cells. The consideration of two levels of staining instead of three clearly improves the performance of the cell classification.

### 4.3 Analysis performance

We compare OIA with other procedures to quantify the immunohistochemical images of oral tissue. The comparison can be made from different points of view: the quality of statistical information provided by the analysis, the expert's analysis time, and the expert's perception.

Once the region of interest is delineated on the image, OIA analyses the positivity for ki67 biomarker^4^ of that region and exports to CSV files the following information: (1) percentage of positive cells; (2) percentage of cells for each staining intensity (high, low, and no stained cells); and (3) distribution of the positivity in the basal, medium, and superior layers of epithelium. These measurements allow clinicians to compare the positivity of ki67 among layers and to study its relationship with the degree of epithelial dysplasia. The clinical practice is implemented in OIA by: (1) analysing the nuclear expression of ki67; and (2) dividing the epithelial thickness into three thirds (layers). This allows comparison of the cell proliferation among epithelium layers, similar to the analysis of epithelial dysplasia (mild-moderate-severe), where architectural and cytological changes are analyzed layer by layer. The basal expression of ki67 can be related to a physiological proliferative activity. In cases of dysplasia, the expression of this biomarker increases and can manifest beyond the basal third.

The OIA software is reliable and precise because it allows to review of the detection and classification of cells before the counting. It is also easy to use and install. Another advantage that the program offers compared to manual quantification is the possibility of sharing the images and results among different experts to evaluate each case, reducing the variability among experts. To the best of our knowledge, there is no other application that automatically counts the cells of each staining level into the three epithelial strata: basal, medial, and superior (see Figure 4). This functionality of OIA allows a global analysis of the epithelium studied, which is of great importance for clinical diagnosis using immunohistochemical techniques, but it is currently not performed in the diary clinical practice due to its difficulty.

OralImmnunoAnalyser runs on a general-purpose computer in a reasonable time. The delineation of the region of analysis and automatic processing takes < 1 min, similar to the Aperio software. The time required for the analysis is dominated by the expert's supervision, which depends on the review needs and the number of cells counted, being about 10 min for counting 1,000 cells (1 min per 100 cells). This revision guarantees that the results are accurate and trustworthy. The traditional procedure to count manually 100 cells in the image takes ~2.3 min. So, OIA represents a saving of 1.3 min for 100 cells, being 2.3 times faster than manual counting and allowing to check the cells counted at any time, which reduces the chances of making human counting errors. Therefore, OIA reduces the analysis time by 56.5% with respect to the manual procedure.

The expert's perception of OIA was evaluated using the system usability scale (SUS), a free questionnaire to measure the learning ability and subjectively perceived usability of computer systems (Bangor et al., 2009; Brooke, 2013). This is a 10-item questionnaire with a five-point scale ranging from 1 (strongly disagree) to 5 (strongly agree), providing a final system score between 0 and 100. The score is calculated by adding up the positively worded items (1, 3, 5, 7, and 9), subtracting one from the user responses, and the negatively worded items (2, 4, 6, 8, and 10), subtracting the user responses from five. Multiplying the SUS score by 2.5 re-scale the score from 0 to 100. A comprehensive interpretation of SUS score (Sauro, 2011) is: SUS < 25 is the worst imaginable system; from 25 to 39 is from the worst imaginable to poor; from 40 to 52 is from poor to OK; from 53 to 73 is OK to good; from 74 to 85 is good to excellent; and above 85 is excellent to the best imaginable system. A small sample of between 8 and 12 users is enough to give a good assessment of how people see the software. The SUS questionnaire to evaluate OIA perception was filled out by eleven experts achieving a mean score of 80.9, which means that the system is from good to excellent from the experts' point of view.

## 5 Conclusions and future work

This work presents OralImmunoAnalyser, a new reliable, and easy-to-use software tool to estimate oral leukoplakia from the immunohistochemical images of mouth tissues. This software combines the automatic detection and classification of cells in the image with a friendly GUI that allows the experts to review the recognition before the calculation of statistical results. OralImmunoAnalyser provides the number of positive (stained) and negative (without staining) cells in the region of analysis, the percentage of cells for each staining level (high, low, and no staining), and the distribution of the cell positivity in different layers of the epithelium (basal, medium, and superior). The software has been tested by the Odontology Faculty of the University of Santiago de Compostela since 2019 in its daily practice. The automatic processing of the images provided the following average performance: (1) the cell detection module achieved a sensitivity of 64.4%, specificity of 93%, and precision of 60.7%; and (2) the cell classification in the three staining levels achieved an accuracy of 79.8%. The time required to analyse each image is dominated by the need for supervision, requiring about 10 min to count 1,000 cells. So, OIA saves 56.5% of time spent by the traditional manual counting of cells, avoiding mistakes because the user can check at any time the cells counted. Despite OIA cannot operate fully automatically, it can considerably accelerate the analysis that can be performed in daily clinical practice, being a major advance over what is currently available. In fact, the expert's perception of OIA achieves a mean score of 80.9 in the SUS questionnaire, which means that the system is from good to excellent.

The OIA software is simple to use and install and has the following advantages: (1) it works with a photograph taken under a microscope and not with a scan of the glass; (2) it allows monitoring, i.e., to see the cells to be accounted for each category before image quantification; and (3) it allows to divide the epithelial strata into three thirds (basal/medium/superior), to compare the positivity of ki67 among layers and to study its relationship with the degree of epithelial dysplasia. For these reasons, OIA is superior to other available tools and its use could be easily implemented in the daily practice of biomedical labs. In addition, this possibility of supervision by the expert favors that OIA can be used as a tool in the teaching-learning process to instruct junior researchers in cell counting.

Although OIA was validated with histological images of oral leukoplakia stained for ki67, our preliminary tests encourage its use with other molecular markers that also stain the cells with brown, such as p53 or p21. This possibility opens up new lines of research that we will address in the future: OIA will facilitate and optimize routinary immunohistochemical analysis and lead to an ever-increasing diagnostic accuracy.

## Data availability statement

The original contributions presented in the study are included in the article/Supplementary material, further inquiries can be directed to the corresponding author.

## Ethics statement

The studies involving humans were approved by Santiago-Lugo Research Ethics Committee, with registration code 2020/470. The studies were conducted in accordance with the local legislation and institutional requirements. The participants provided their written informed consent to participate in this study.

## Author contributions

ZA-T: Data curation, Formal analysis, Investigation, Methodology, Software, Validation, Visualization, Writing – original draft, Writing – review & editing. MP-C: Data curation, Investigation, Methodology, Validation, Visualization, Writing – original draft, Writing – review & editing. EC: Conceptualization, Formal analysis, Methodology, Project administration, Resources, Software, Supervision, Writing – review & editing. JS-P: Resources, Validation, Writing – review & editing. MF-D: Formal analysis, Investigation, Methodology, Writing – review & editing. AM: Data curation, Methodology, Writing – review & editing. MG-T: Resources, Supervision, Validation, Writing – review & editing. PG-V: Conceptualization, Investigation, Methodology, Project administration, Resources, Supervision, Validation, Visualization, Writing – review & editing.

## Footnotes

^1^ https://www.leicabiosystems.com/

^2^ https://www.gtk.org/

^3^ https://opencv.org/

^4^Positive cells are those stained of brown color independently of its intensity.
