# Supplementary material for: OralImmunoAnalyser: a software tool for immunohistochemical assessment of oral leukoplakia using image segmentation and classification models
Source: Front Artif Intell. 2024 Feb 26;7:1324410. doi: 10.3389/frai.2024.1324410 (PMC10925674; doi:10.3389/frai.2024.1324410)
Supplement: Supplementary file 1 [file Data_Sheet_1.PDF]

frontiers

Supplementary Material

1 WHAT IS ORALIMMUNOANALYSER?

OralImmunoAnalyser is a free software tool to quantitative analyse oral immunohistochemistry images of patients. It is a multi-platform software written in C/C++ programming language. It was developed by the Research center of Intelligent Tecnhologies (CiTIUS) [1] in colaboration with the Stomatology Department of Medicine and Odontology Faculty, both belonging to the University of Santiago de Compostela (Spain). OralImmunoAnalyser (OIA) examines automatically the immunohistochemistry images using advanced computer vision and machine learning techniques. But, sometime the automatic processing is not satisfactory to the expert. So, OralImmunoAnalyser includes a friendly GUI (Graphical User Interface) to review, draw, interact and visualize data.

OralImmunoAnalyser work with three types of files: image files and the text files XML (eXtensible Markup Language) and CSV (Comma-Separated Values). The image formats supported are the most frequently used formats like GIF, TIF, PNG, BMP, PPM, JPG, etc. There is one XML file per image to save the quantitative analysis, which includes the region of analysis and the staining level of the cells in the region. This information is overlapped on the image and it is frequently called overlays. The CVS file contains the statistical information of the quantitive analysis of the image. So, OralImmunoAnalyser manages in different files the image, the overlays with the analysis and the results. This organization allows that the experts can load, share or review an analysis every time later. The software counts the number and percentage of cells for each staining level (highly stained, low stained and without staning) in the region of analysis, and provides also these measures for different subareas in the region of analysis (basal, medial and superior areas).

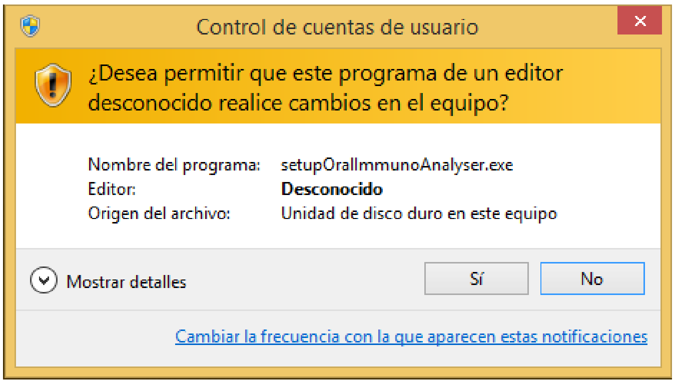

Figure S1. Window to install OralImmunoAnalyser.

The current user guide is organized as follows: sections 2 and 3 describe the installation steps for Windows and Linux operating systems respectively; section 4 describes the editing tools of the Graphical User Interface (GUI) of OralImmunoAnalyser. Section 5 describes the configuracion of the working preferences. Sections 6, 7 and 8 describe the functionality of File, Edit and View menus, respectively. The section 9 describes how to do the quantitative analysis of one image. Section 10 describe the statistical

[1] http://citius.usc.es/

results provided by the software. Section 11 describes the issues related to the classification of the stained level of cells. Finally, Section 12 refers to Menu Help.

2 WINDOWS INSTALLATION

The installation process is the common process to install programs in Windows. First, do double click on the file `setupOralImmunoAnalyser.exe`. As OralImmunoAnalyser (OIA) is a foreing program, you need to set permisions to allow the installation of foreing programs. Then, it is open a window asking us about to allow to install a foreing program `setupOralImmunoAnalyser.exe` on your computer. You must click "Yes" in figure S1 to allow the installation of the program. After clicking "Yes", the window of Figure S2 is open. If you click "Next", the program go on installing.

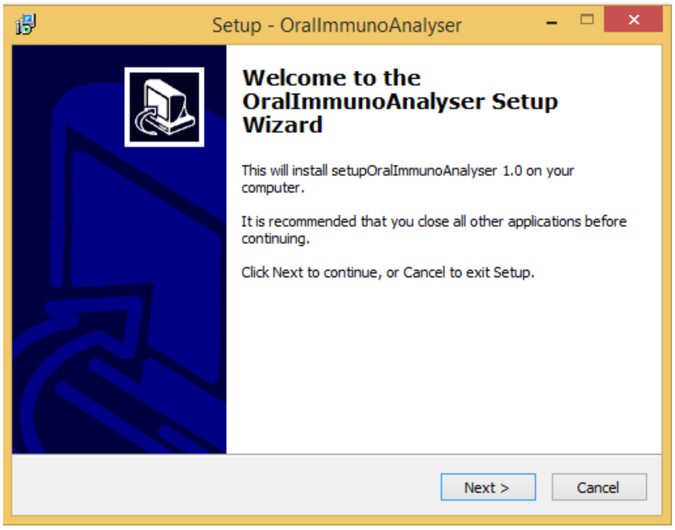

Figure S2. Window to install OralImmunoAnalyser.

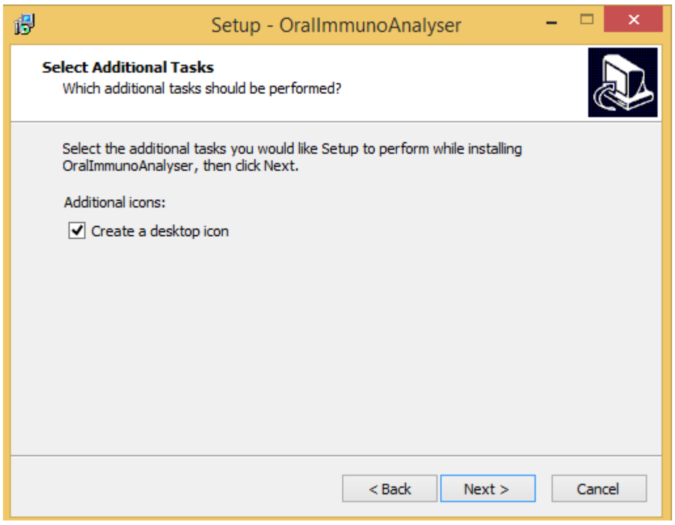

Figure S3. Window to install OralImmunoAnalyser. Click on the check box to create a direct access in the desktop.

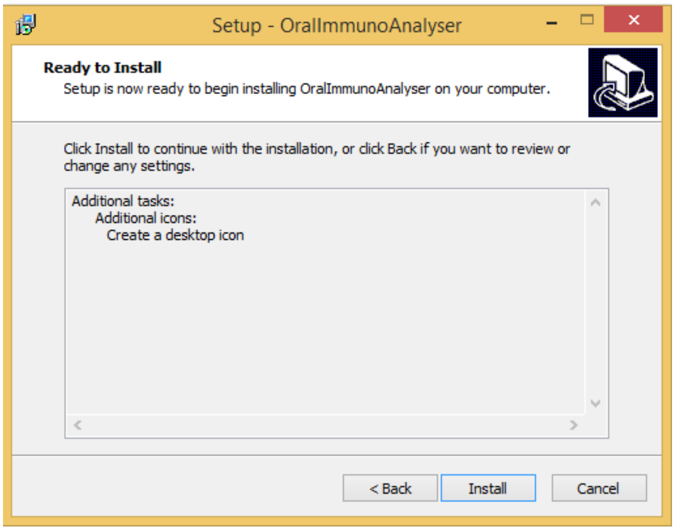

Figure S4. Window to install OralImmunoAnalyser. Ready to install OralImmunoAnalyser.

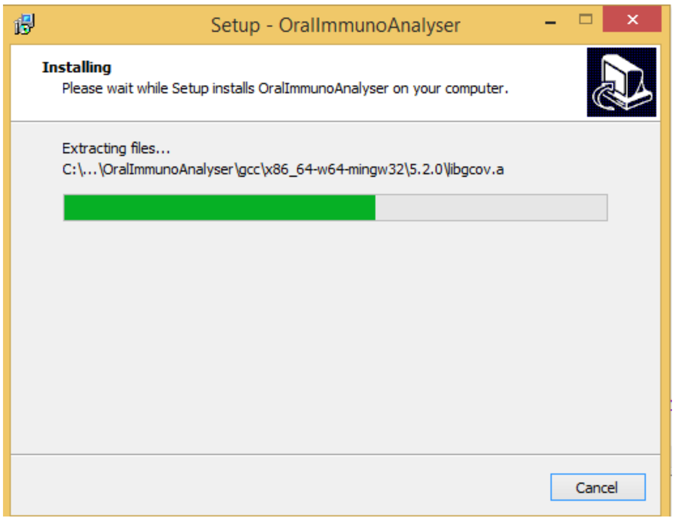

Figure S5. Window to install OralImmunoAnalyser. Installing OralImmunoAnalyser.

The next installation window is shown in Figure S3, which asks if you want to create a direct access icon in the desktop. We recommend you activate the check box because it will be faster the access to OralImmunoAnalyser. Then, you click on the button "Next" to go to the next window (Figure S4). It is a window to confirm if you want to install OralImmunoAnalyser (clicking with the mouse on button "Install") or cancel the installation (clicking the button "Cancel").

After starting the installation process, it will pop up a dialogue showing the installation process, as it can be seen in the Figure S5. This process can take a few seconds. If you click on the button "Cancel", the installation process will be cancelled. When the installation process finished, it will be shown the window of Figure S6. In this window, you click the "Finish" button to finish the installation process. If the check box *Lunch OralImmunoAnalyser* is marked, OralImmunoAnalyser will be run. Otherwise, OralImmunoAnalyser will not run now, but you can run every time later double clicking the desktop icon of OralImmunoAnalyser, which was created in the desktop during the installation process.

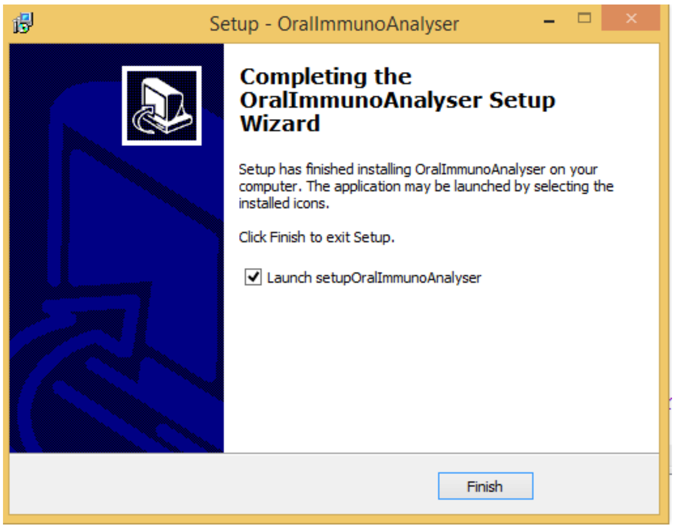

Figure S6. Window to install OralImmunoAnalyser. Completing the OralImmunoAnalyser Setup Wizard.

3 LINUX INSTALLATION

In Linux, there are several types of packages, and every distribution has its own preferred package format. Ubuntu distributions used the Debian packages (format DEB). It is in construction.

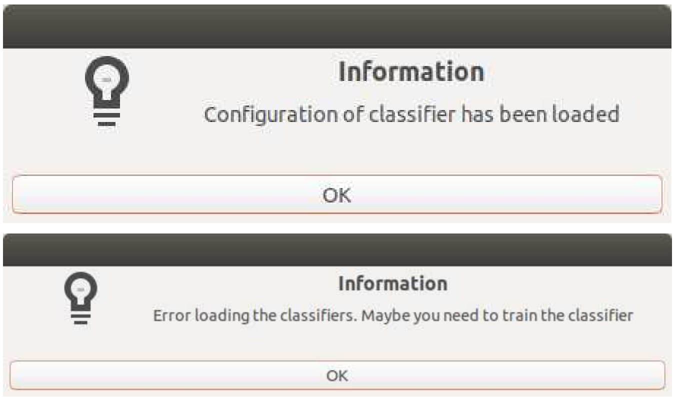

Figure S7. Pop-up windows show when OIA is run to inform if the classifier is loaded or not.

4 RUN ORALIMMUNOANALYSER

After installing OralImmunoAnalyser (OIA) in your computer using Windows, double click on the desktop icon of OralImmunoAnalyser to run it. OIA checks if the classifier was loaded. If there is a classifier trained in the computer, the pop-up message of upper panel of figure S7 will be shown, otherwise, it is shown the pop-up message of lower panel of figure S7, which informs that the classifier must be trained before classifing cells (see the section 11 for the details of classifier). After clicking **Ok** in the window, the main window of Figure S8 will be open. It encloses a menu bar (at the top of screen) containing all menu commands, a toolbar (under the menu bar) containing tools to access to the main functionality of OralImmunoAnalyser, and an image window (at the bottom of screen) in which the

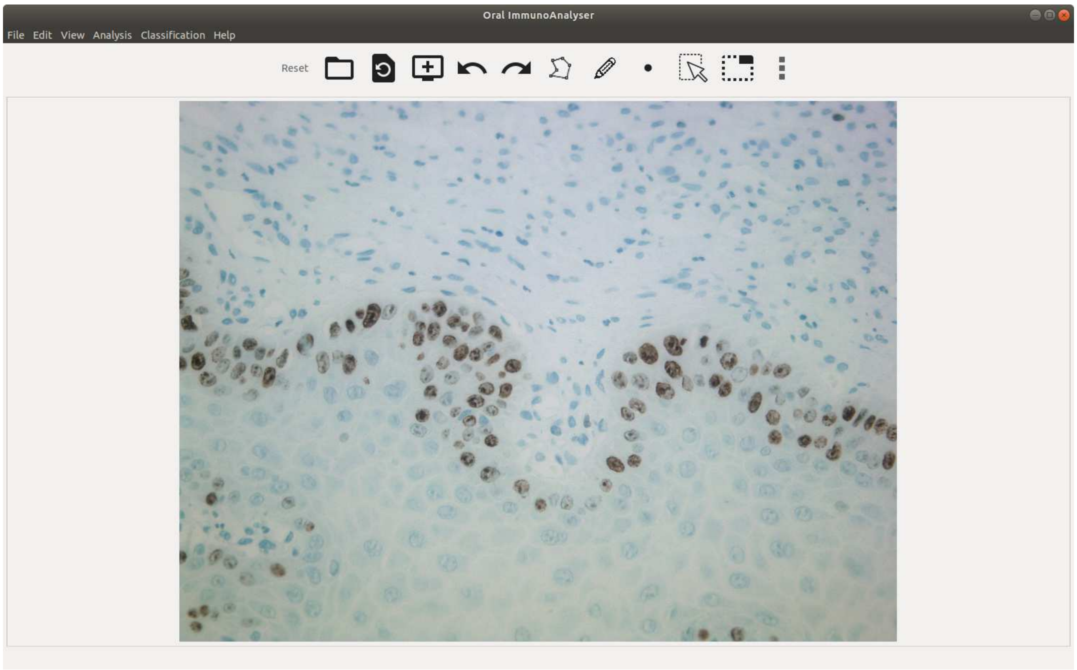

Figure S8. Main window of OralImmunoAnalyser.

immunohistochemistry images are open. The menu bar lists all **OralImmunoAnalyser** commands. It is organized in six menus:

1. **File**: Basic file operations (opening images and XML files, saving the overlays on the image in files and statistical results); set and load the working preferences; and exit of OralImmunoAnalyser.
2. **Edit**: redo and undo operations, fit the image to the window size and set the image to the original size.
3. **View**: show or hide the processing panel, also called lateral panel.
4. **Analysis**: provides functionalities to calculate the results of many images jointly.
5. **Classification**: provides functionalities to classify cells into their stained level (high stained, low stained, without staining) and to train the classifier.
6. **Help**: the help functionality.

The toolbar is a fast access to the main functionality of OralImmunoAnalyser, which contains the following icons (if the mouse is put on the icon, a pup up message showing the functionality of the icon is open):

1. **Reset**: (first icon) clear all the objects drawn or overlayed on the image.
2. **Open** : (second icon) open a dialog to select the immunohistochemistry image to be open (see section 6).
3. **Zoom Fit**: (third icon) fit the image zoom to the image window.
4. **Original Zoom**: (fourth icon) set the original image zoom.
5. **Undo**: (fifth icon) when you click this button, undo the last overlay deleted on the image (operate only with the overlays manually deleted).

6. **Redo**: (sixth icon) when you click this button, redo the overlays deleted (operate only with the overlays manually deleted).
7. **Draw with points**: (seventh icon) activate the draw of regions tool. While this button is pressed, the user draws a region marking points with the left button of the mouse and finishs the outline of the region when the user click the midle button of the mouse.
8. **Draw freehand**: (eighth icon) activate the draw freehand regions tool. When this button is pressed, the user can draw a freehand region pressing the left button of mouse and keeping it pressed while you are drawing the region. When you release the mouse button, the region is finished.
9. **Draw points**: (nineth icon) when this button is activated, you can mark points, which can represent points of a region, basal point or cells (as the software OIA considers the cells as points), on the image clicking the left button of the mouse.
10. **Select**: (tenth icon) when this button is activate, you can select an object drawn on the image. The object is selected clicking into the region (for select regions) or near the point (to select points) with the left button of the mouse. To select more than one object, keep the key **Ctrl** or key “Control” pressed while selecting objects.
11. **Select with rectangle**: (eleven icon) when this button is activate, you can select many objects drawn on the image drawing a rectangle. For that, click the left button of the mouse and keepping the button pressed, drag the mouse to draw a rectangle and release the left button. The objects inside the rectangle are selected.
12. **Lateral panel**: (twelfth icon) open the lateral panel, which will be used to process and analyse the open image (see figure S9).

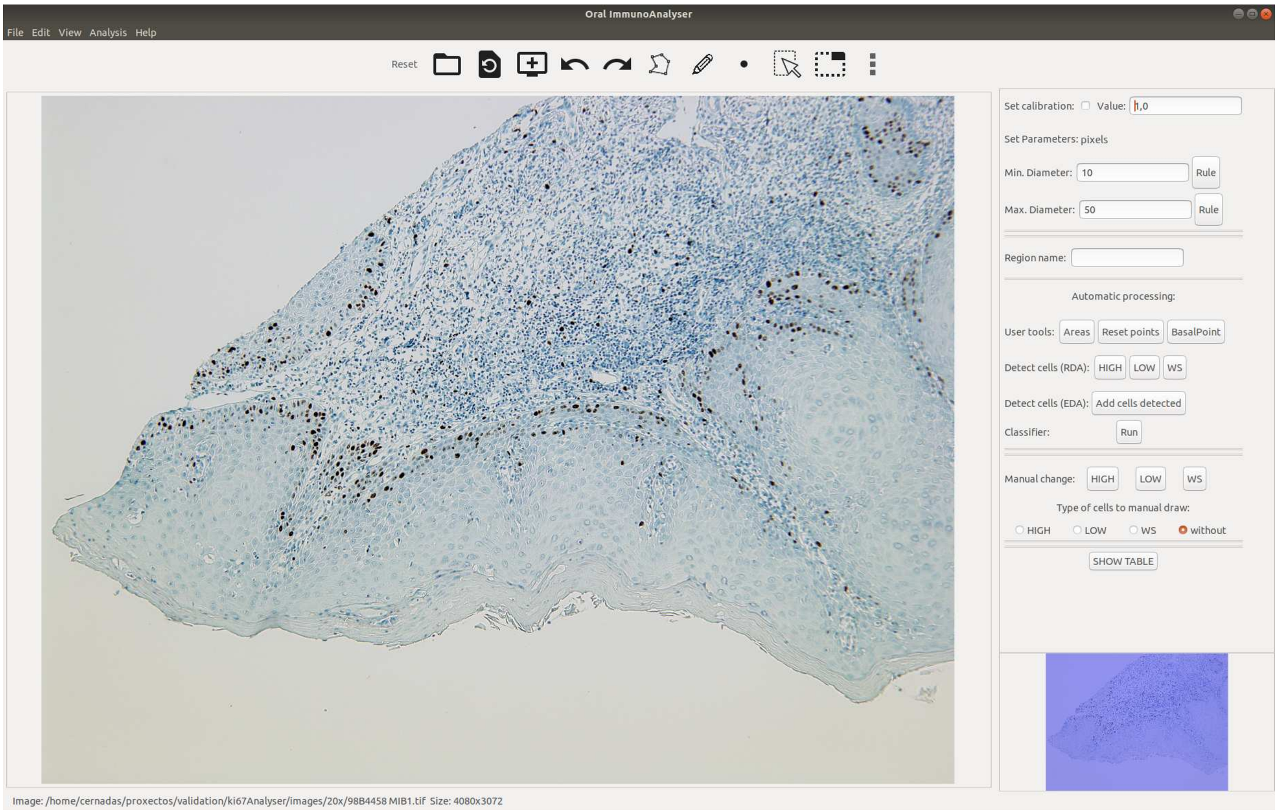

Figure S9. Main window of OralImmunoAnalyser with a typical immunohistochemistry image loaded and the lateral panel open.

In the following sections, the functionality of the Graphical User Interface (GUI) of OralImmunoAnalyser will be described. The submenus of the File menu from the menu bar are:

1. **Open image**: open a dialogue window to load a new image.
2. **Open image and XML**: open a dialogue window to select the image to load. But, if the XML file for that image exists, its overlays are also loaded and drawn on the image. To do this process, the XML file must be named with the same name as the image and it is in the XML folder specified in the working preferences.
3. **Open XML**: open a dialogue window to select the XML file and put its content on the image loaded. The user controls that this XML file is suitable to the image loaded in OralImmunoAnalyser.
4. **Save XML**: open a dialogue window to select the name of XML file in which you want to save the overlays drawn on the image. By default, OralImmunoAnalyser sets the name of the image file, but with extension XML, and it is stored in the XML folder specified in the working preferences.
5. **Export CSV**: open a dialogue window to select the name of CSV file in which you want to save the statistical analysis of the image. By default, OralImmunoAnalyser sets the name of the image file, but with extension CSV, and it is stored in the CSV folder specified in the working preferences.
6. **Preferences**: open a dialogue window to set our working preferences in OralImmunoAnalyser (working directories, colour and width of lines, calibration and diameters). The working preferences can be saved in the computer to be available in next sessions.
7. **Reset preferences**: reset all the OralImmunoAnalyser preferences set previously in the computer.

8. **Load preferences**: internally, the preferences are saved in a XML file. This option allows to load the working preferences from an external XML file.
9. **Exit**: exit of the program.

Section 5 describes the configuracion of the working preferences in OralImmunoAnalyser.

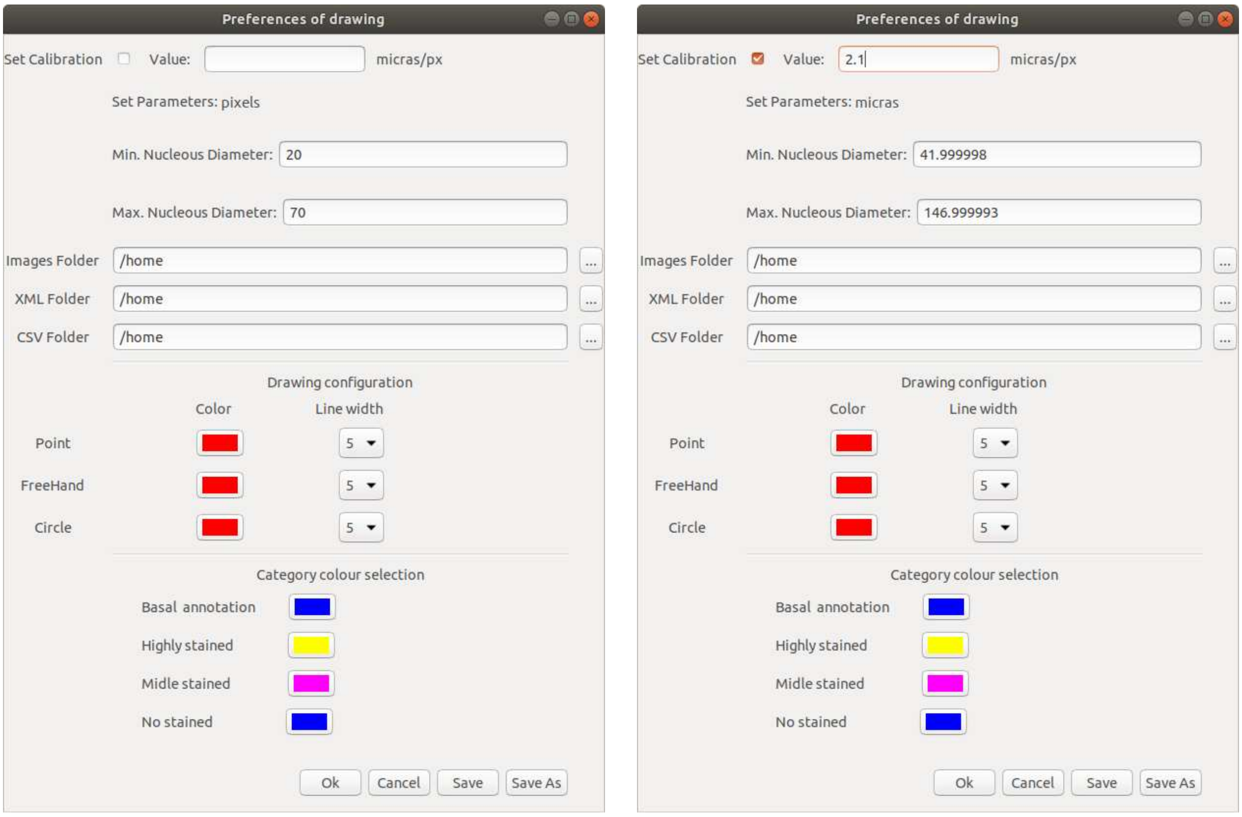

Figure S10. Window to configure the preferences of OralImmunoAnalyser: default configuration (left panel) and after setting the calibration (right panel).

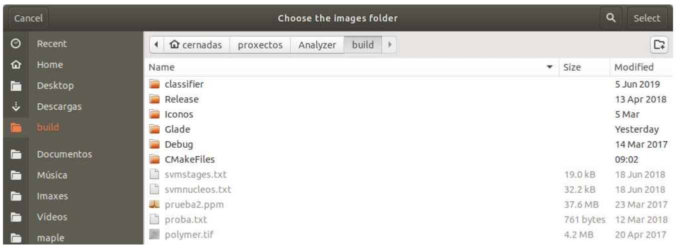

Figure S11. Window to choose a working directory.

5 SET PREFERENCES

Selecting the submenu "Preferences" of menu File, the window shown in Figure S10 (left panel) will be open. The items to configure are:

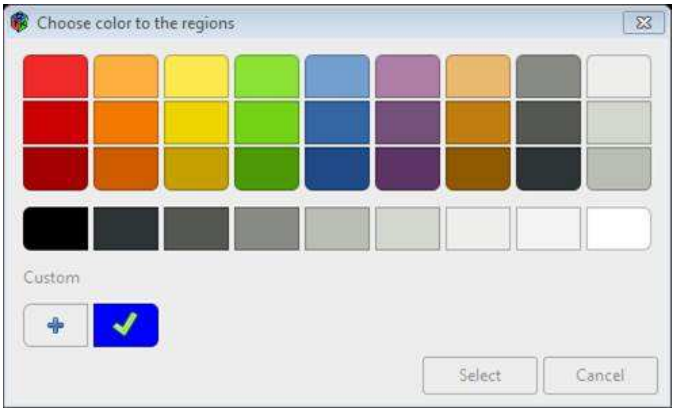

Figure S12. Window to choose the colour to draw cells on the image.

1. **Calibration**: it is the number of micrometers per pixel in the image, which depends on the image acquisition system. It allows to set the units to measure the objects (region of analysis in our case).
2. **Diameters**: it allows to put the minimum and maximum size of the cells in the images. These parameters can be set in pixels (if the calibration is not set) or micras (if the calibration is set).
3. **Working directories**: it allows to set the default directories of the images, XML files and CSV files to use OralImmunoAnalyser.
4. **Drawing configuration**: it allows to set the colour and line width preferences to draw the objects overlapped to the images.
5. **Category colour selection**: it allows to set the colour to visualize different categories in OralImmunoAnalyser. The categories used by the software are: 1) the stained level of the cells, which we consider "high stained", "low stained" and "without staining"; and 2) the colour to visualize the basal point on the image, which is set using the button **BasalPoint** in the lateral panel (see section 9.2 for more details).
6. **Set or change the configuracion**: the buttons **Ok**, **Cancel**, **Save** and **Save As** at the bottom of Figure S10 set, cancel or save the configuration.

5.1 Calibration

The top of Figure S10 shown a check box after the label *Set Calibration*. This check mark assures that the Calibration real value has been added, if it is non-checked, then the calibration is considered in pixels. After the label *Value*, and if the check box of calibration is active, you can set the number of micras or micrometers per pixel in the image (press the "Enter" key after put the value). The calibration value are fixed by the digitalization process, depending on the magnification used in the microscope and the spatial resolution of the digital camera connected to it. The right panel of Figure S10 shows the left panel after activating the calibration and set its value to 2.1 micras per pixel. As it can be seen comparing both panels in Figure S10, when the check box of calibration is modified, the units and values of diameters change.

5.2 Diameters

The automatic detection of cells in OralImmunoAnalyser improves if the user provides the minimum and maximum diameter of the cells that the user want to detect. These parameters can be set after the labels *Min. Diameter* and *Max. Diameter* respectively, puting the values of minimum and maximum diameter (in micrometers if the calibration is active and in pixels if the check box of calibration is not

marked). Obviously, if the minimum diameter set by the user is higher than the maximum diameter or the maximum diameter set is lower than the minimum diameter, the value of diameter is not updated. After set each diameters, you must press the “Intro” or “Enter” key to update the value in OralImmunoAnalyser. The minimum and maximum diameters of the cells to recognize can also be set on the lateral panel as text or graphically (see section 8).

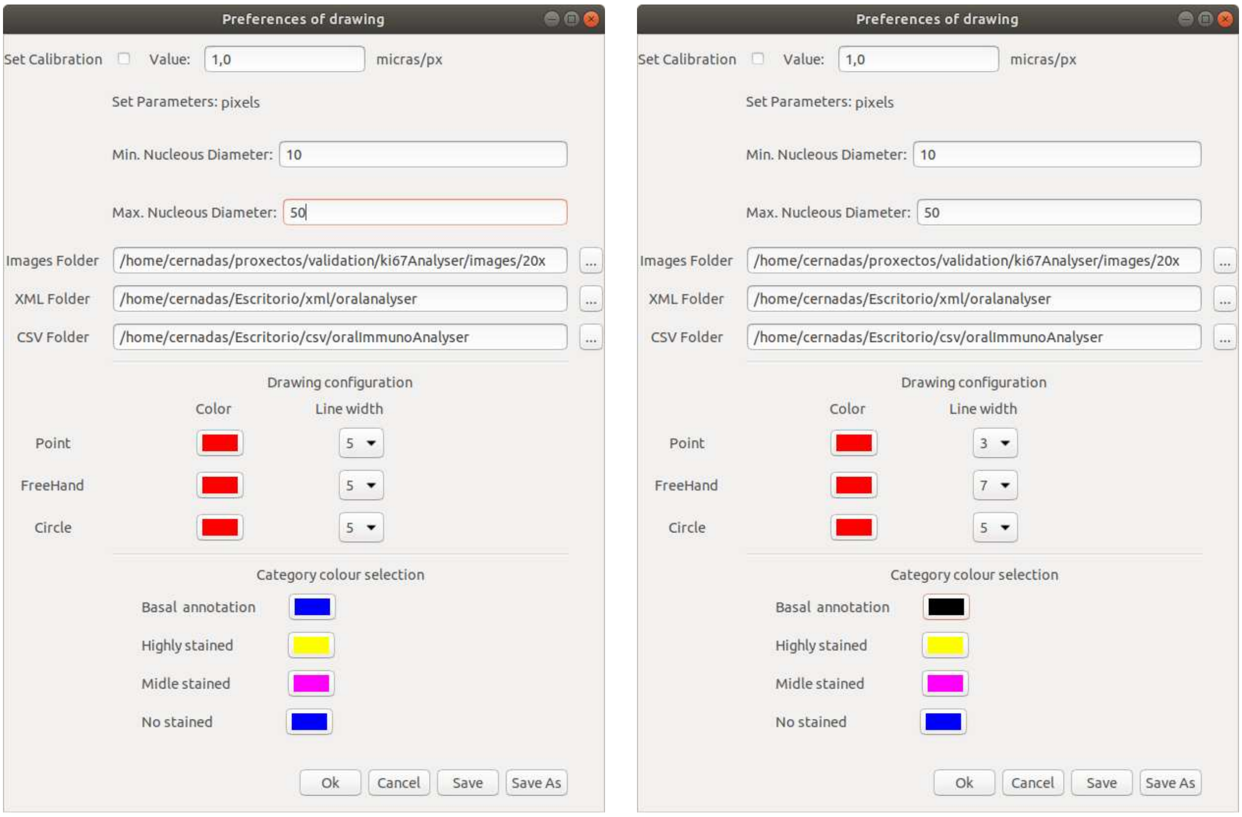

Figure S13. Window of preferences of figure S10 after: setting the working directories (left panel) and setting the drawing configuration and categories colour (right panel).

5.3 Working directories

After the labels *Images Folder*, *XML Folder* and *CSV Folder* in Figure S10, there are their correspondent entry widgets to visualize the default directory to store the images, XML files and CSV files respectively. After each entry widget there is a button with three points. Clicking this button open the folder chooser dialogue of Figure S11 to choose the work directory for images and the left panel of Figure S13 shows the preferences window after modifying the working directories and the diameters.

5.4 Drawing configuration

You can change the colour and line width to draw the regions and points (cells) overlapped to the image. Clicking with the mouse the red button after the label *FreeHand* or *Point* opens the colour chooser dialogue shown in Figure S12. Selecting a colour in this window and pressing the button “Select”, you change the drawing colour to freehand or point objects. Clicking in the button after the colour button, a drop-down list is open to select the line width to the freehand or point object. Points are shown as a hole circle in order to visualise the cells.

5.5 Category colour selection

As in the section 5.4, clicking the colour button after the labels of the fundamental categories: basal point, hight stained, low stained and no-stained open the colour chooser dialogue, in which you can change the colour to represent that categories in OralImmunoAnalyser. Figure S13 shows the figure S10 in which the width and colour are set for some categories.

5.6 Set or change the configuration

The four buttons **Ok**, **Cancel**, **Save** and **Save As** at the bottom of Figure S10 have the following functionalities:

1. Clicking the button **Ok**, you set these preferences to the present working session.
2. Clicking the button **Cancel**, you cancel the operation of setting the preferences and the preferences will not be updated.
3. Clicking the button **Save**, you set these preferences to the present working session and to the following working sessions in the future.
4. Clicking the button **Save As**, you set these preferences to the present working session and OralImmunoAnalyser saves these preferences in a XML file selected by the user, which can be loaded every time using the submenu "Load preferences" of menu File in this computer or other one. This option allows to share the working preferences with other expert (it may be possible that the working directories do not work correctly).

6 FILE MENU

6 FILE MENU

The items available within **File** menu are: **Open Image**, **Open Image and XML**, **Open XML**, **Save XML**, **Export CSV**, **Preferences**, **Load Preferences** and **Exit**. The **Exit** menu or the **X** button in the top-right side of window quit OralImmunoAnalyser. As mentioned, OralImmunoAnalyser works with three types of files: image files, XML (eXtensible Markup Language) and CSV (Comma-Separated Values) files. There is one XML file per image, which saves the contour of region of analysis with the cells (visualize as points in the image window) detected, in order to allow that this analysis can be loaded into the software in other instant of time. The CVS file exports the statistical information of the quantitave analysis of the image.

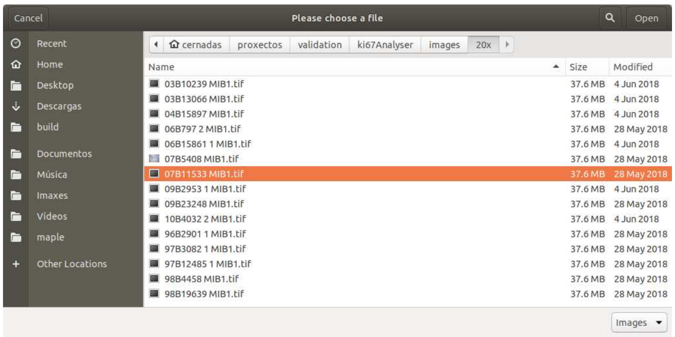

Figure S14. File chooser dialogue to choose the image to be loaded in OralImmunoAnalyser.

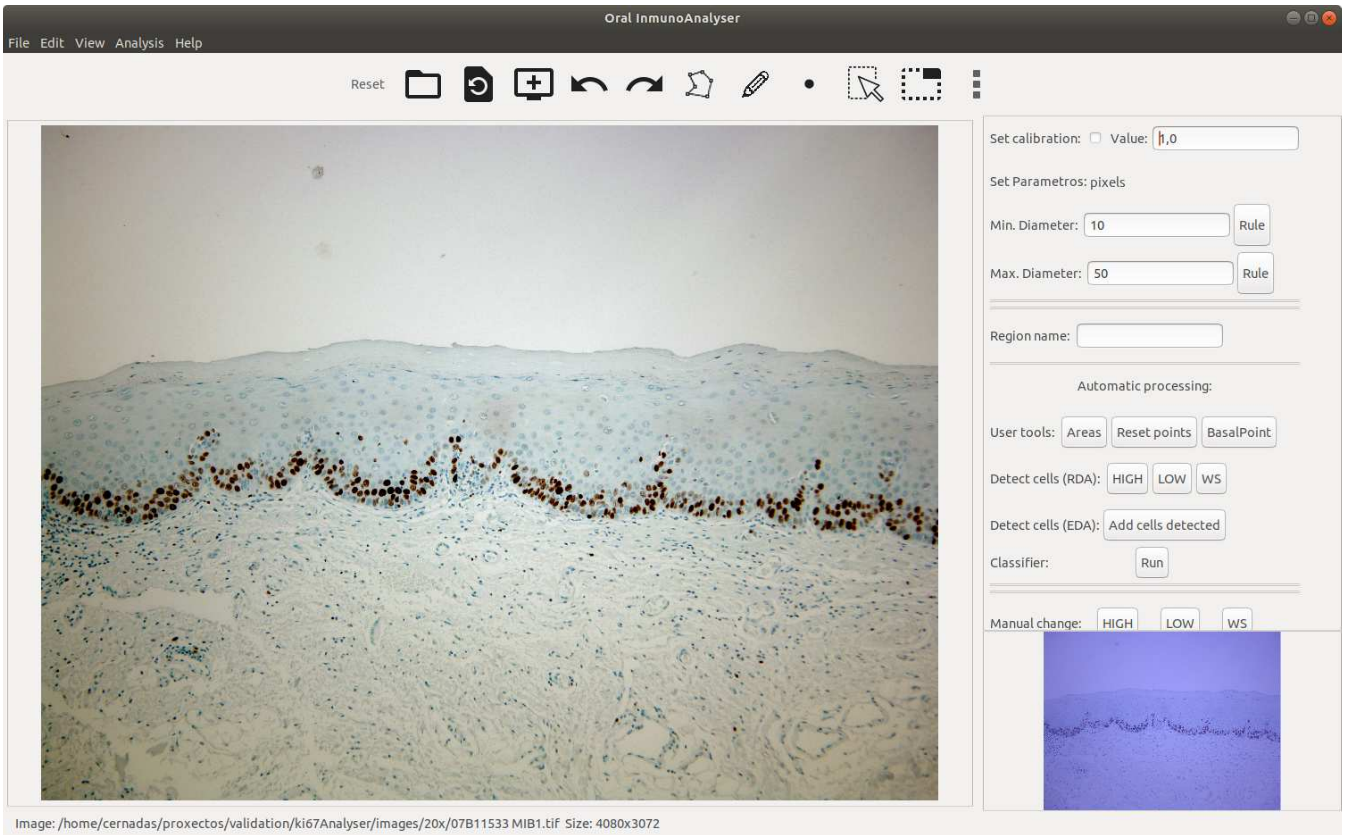

Figure S15. The image selected in Figure S14 is loaded in OralImmunoAnalyser.

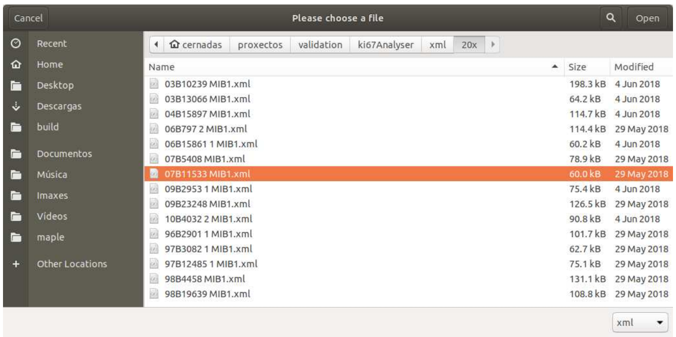

Figure S16. File chooser dialog to choose the XML file to be loaded in OralImmunoAnalyser.

The **Open Image** submenu or the second button of the toolbar opens the file chooser dialogue of Figure S14. The directory open is the one set in the configuration of the preferences (see the section 5). Choose the path to the image and click the button **Open** in the bottom of the window to load the image in OralImmunoAnalyser. Take care that the image path is not biggest than 256 characters or the image path contains rare symbols, because OralImmunoAnalyser could not work correctly. Figure S15 shows an image loaded in OralImmunoAnalyser. When an image is loaded, the lateral panel is open and in the bottom of the main window appear a state bar with the name of image and its size.

If the image was analysed in other instant and the XML file was stored, you can load this XML file and overlap to the image selecting the submenu **Open XML**. This operation opens a file chooser dialogue to choose the XML file (see the Figure S16) and overlap the region outline and cells to the image, as can be

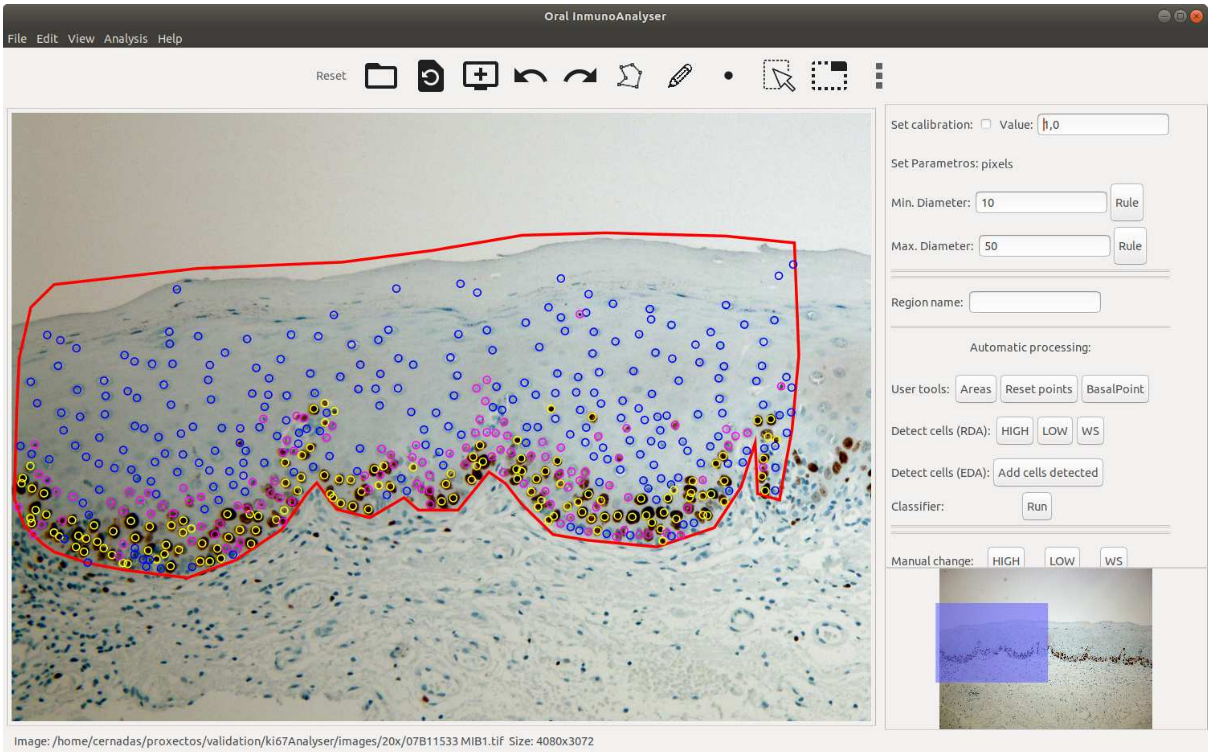

Figure S17. OralImmunoAnalyser shows the image of figure S15 with the date of XML file selected in figure S16 overlapped.

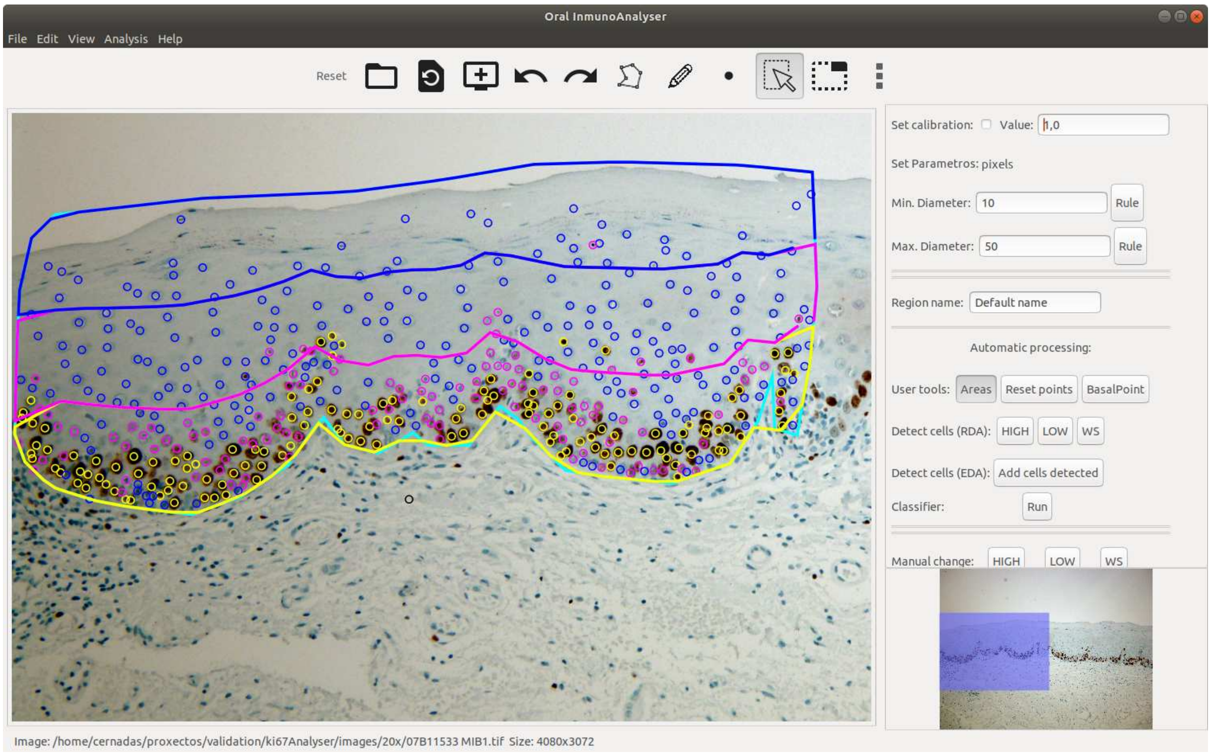

Figure S18. OralImmunoAnalyser shows the image of figure S17, in which the calculation of basal, medial and superior areas are visualized.

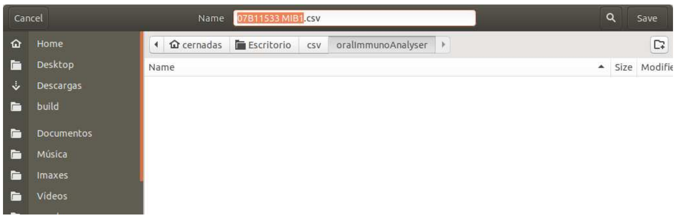

Figure S19. File chooser dialog to choose the CSV file to store the statistical analysis of the image.

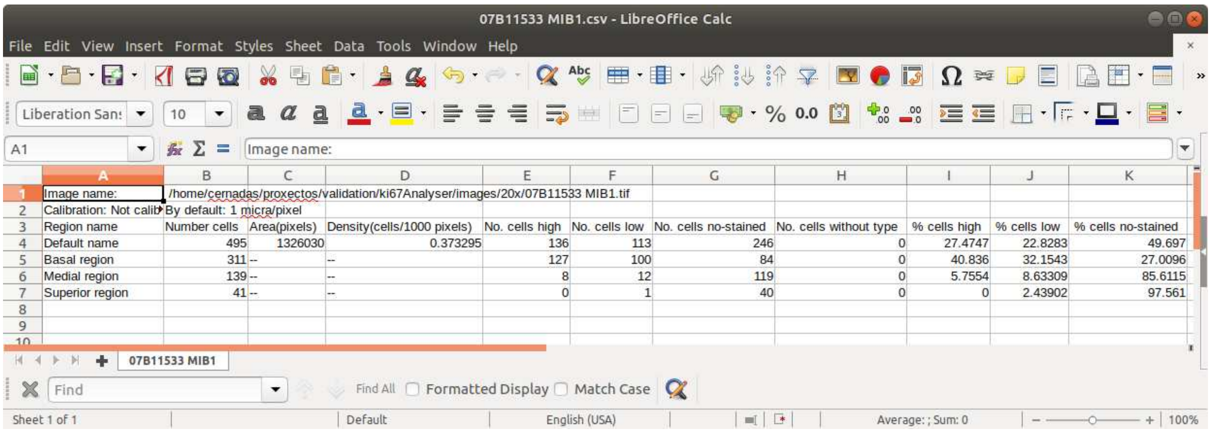

Figure S20. An example of CSV file of the image of Figure S18 imported in LibreOffice Calc.

seen in Figure S17. The user must select the right XML file for each image, OralImmunoAnalyser does not check if this XML file corresponds to a specific image file.

The above process can be done in one step with the submenu **Open Image and XML**, which open a file chooser dialogue, as in Figure S14, to choose the image to load and OralImmunoAnalyser checks in the XML path set in the preferences if there is a XML file with the same image name and extension `.xml`. In this case, OralImmunoAnalyser opens this XML file and overlaps its content over the image loaded (go directly to figure S17).

Once the image was analysed (automatically or manually), the statistical analysis can be saved in the CSV file. Figure S18 shows the basal, medial and superior regions (see section 9.2 to a further description), which are visualized if the toggle button **Areas** in the lateral panel was activated and the basal point was marked by the user. The submenu **Export CSV** opens the file chooser dialogue in the CSV directory selected in the preferences (see figure S19) to select the name of CSV file (by default it is used the name of image with the extension CSV). OralImmunoAnalyser stores the statistical analysis of the image in that file. The information stored is: image path, calibration used, total number of cells, area of the region of analysis (in pixels if calibration is not set and in micrometers otherwise), density of cells in the region of analysis, number of cells high stained, number of cells low stained, number of cells without staining, number of cells that the user do not assign any category, percentage of high stained cells, percentage of low stained cells and percentage of cells without staining. If the basal point was marked by the user, the number of cells of each category and their percentages for the basal, medial and superior area are provided. The information stored in the CSV file can be loaded in a spreadsheet as LibreOffice Calc[2], as it can be

[2] `https://www.libreoffice.org/discover/calc/`

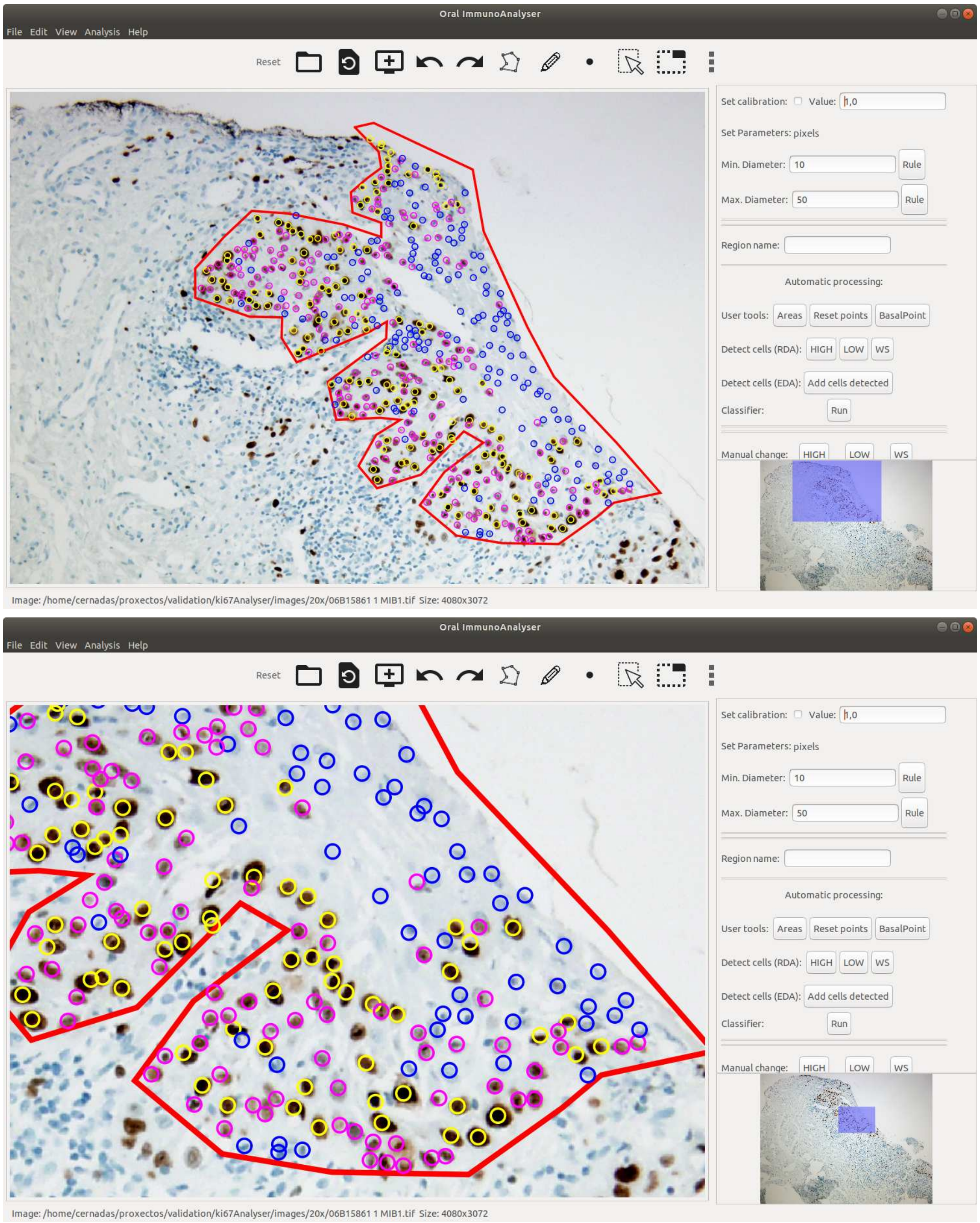

Figure S21. A zoom of the image in OralImmunoAnalyser.

seen in Figure S20 (take care that the decimal numbers are represented by a point in the CSV file and not by comma).

The **Load preferences** submenu opens a file chooser dialogue to select the XML file preferences (`preferences.xml` file) to load in the computer.

7 EDIT MENU

The items available within this menu are also available in the toolbar (see section 4). They are: **Undo** (fifth icon in the toolbar), **Redo** (sixth icon in the toolbar), **Fit Image** (third icon in the toolbar) and **Original Size** (fourth icon in the toolbar).

The **Fit Image** item fits the image zoom to the image window (for example see the figure S9) and the **Original Size** sets the original image zoom. Another image zoom can be achieved rolling up the mouse wheel to increasse the zoom and rolling down the mouse wheel to decreasse the zoom. If the lateral panel is open, the position of the visible image area in the image window can be seen in the icon image located in the bottom of lateral panel (see Figure S21). Keeping this zoom, you can move to another part of the image by two methods: 1) pressing simultaneously the left and right buttons of the mouse and move the mouse to displace the visible area; and 2) press the left mouse button on the blue square in the icon image of the lateral panel and move it. Both the visible area in the window image and the icon image of lateral panel are synchronized. As can be seen, the overlays of the image are zoomed with the image.

8 VIEW MENU OR LATERAL PANEL

The only item available is **Processing Panel** (also included in the last icon of the toolbar), which closes or opens the lateral panel of figure S9. The lateral panel contains the following functionality from top to bottom:

1. **Calibration** (first line): shows the calibration preferences set in the software or allows to change the calibration options as in the configuration preferences window (see section 5).
2. **Diameters** (third and fourth line): shows the minimum and maximum diameters set in the preferences of the program or allows to set these values. The values of the minimum or maximum diameter can be set numerically, using the entry widgets (as it was described in section 5), or graphically, using the button **Rule**. When the calibration or diameters are modified, all the overlays of the image are deleted. Using the entry widgets, the value of the diameter would be provided in pixels, if calibration is not activated, or in micrometers otherwise. To choose the diameter graphically, do the following steps: 1) click on the **Rule** button (the button remains activated); 2) draw a line on the image window pressing the left mouse button and when you move the mouse, with the left mouse button pressed, the line is drawing; and 3) when you release the left mouse button, the **Rule** button will become desactivated and the length of the line is put in the entry widget (this value does not appear if the minimum diameter is superior than maximum diameter or the maximum diameter is lower than the minimum one). The changes done in the lateral panel for calibration and diameters are only applied for the current working session. But, if you want to save permanently these changes you must go the preferences submenu (see section 5).
3. **Region name** (fifth line): after this label widget, there is a entry widget in which the user can assign a name (text) to the selected region. The software assigns the name “Default name” by default.
4. **Automatic processing**: (from seven to ten line) after this label, there is a set of four lines to automatically process the image, which will be described further in section 9.
5. **Category asignment**: (from eleven to thirteen line): there are a set of functionalities to manage the issues related to the assigment of categories to the cells detected (see also the 9 section).

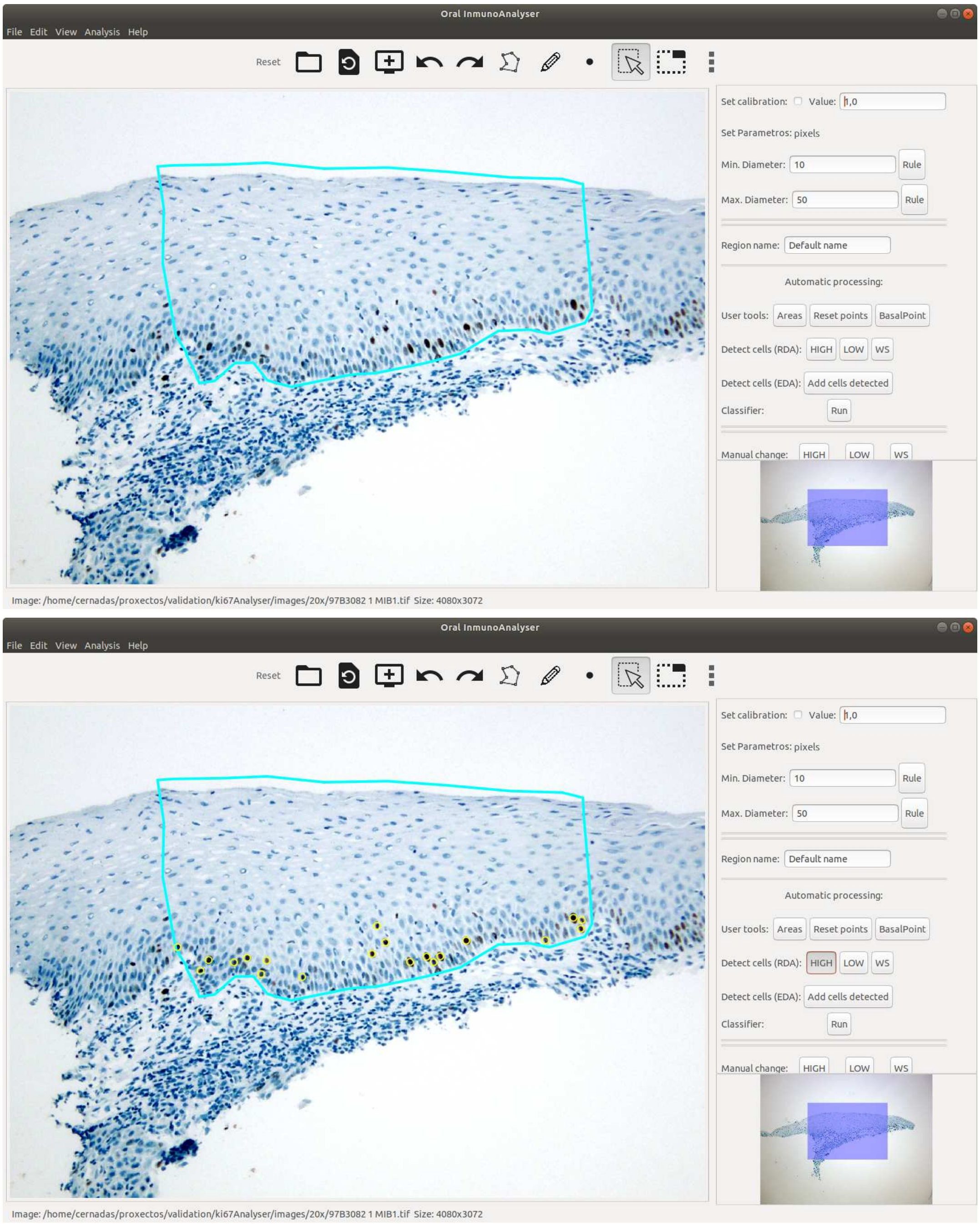

Figure S22. After a region of analysis was drawn and selected (upper panel) and the upper region after the use the button **HIGH** of RDA method (lower panel).

6. **Visualization of results**: when pressing the toggle button **Show Table**, a table containing analysis information is open in the bottom (more details in section 9). If the table is open, you can click that button (now with the message **Close table**) to close the table.
7. **Visualization position**: at the bottom of the lateral panel, there is a miniature or icon image of the original image loaded in OralImmunoAnalyser. Over this miniature, there is overlapped a blue shadown square showing the part of the original image, which is shown in the image window. The

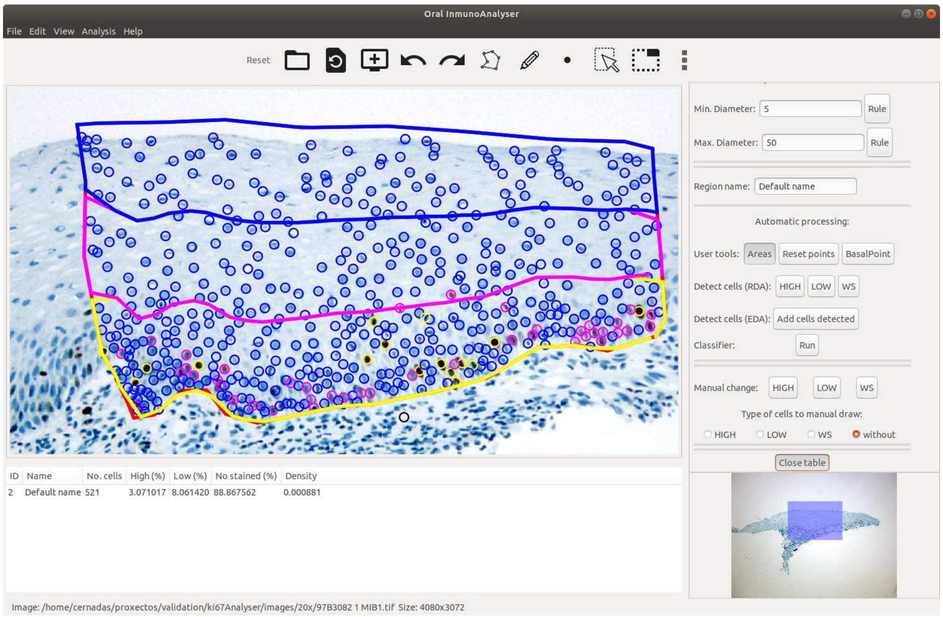

Figure S23. Visualization of information of the image analysis in the bottom table (after clicking the button **Show Table** in the lateral panel).

position and size of this blue square depends on the zoom used in that moment, as can be see in figure S21.

9 AUTOMATIC PROCESSING

To process automatically an immunohistochemistry image, the user must do the following steps, pointing out if that step is required or optional:

1. **Draw the region of analysis** (required): the user must manually draw the region of analysis. So, activate the freehand button in the toolbar (seven or eight button) and draw a region in the image window using the mouse. Finally, activate the button **Select** (tenth button in toolbar) and click inside the region with the left button of the mouse to select the region (see upper panel of figure S22).
2. **Change the name to the region** (optional): by default the name of the region is “Default name”. The user can change this name writting a new text in the entry widget after the label *Region name* in the lateral panel.
3. **Detect cells inside the region** (required): detect automatically the cells inside the selected region using the buttons of lines eight and nine of lateral panel. A further description is in section 9.1.
4. **Classify cells** (required): once the cells are automatically detected, they must be classified to assign a category to each cell. The categories included in OralImmunoAnalyser are: 1) cells high stained; 2) cells with low stained; and 3) cells without staining (further details in section 9.1).
5. **Manual supervision** (optional): as the immunohistochemitry images are very complex and exist a high variance among images, the automatic processing is frequently incomplete or not perfect. Every time the user can use the edition tools provided by the GUI to delete and add cells. The user can also change the cell category using the buttons **HIGH**, **LOW** and **WS** after the label *Manual change* (line eleven in the lateral panel). To change the category of a cell or set of cells click in the suitable button taking in mind that the cells must be selected previously.

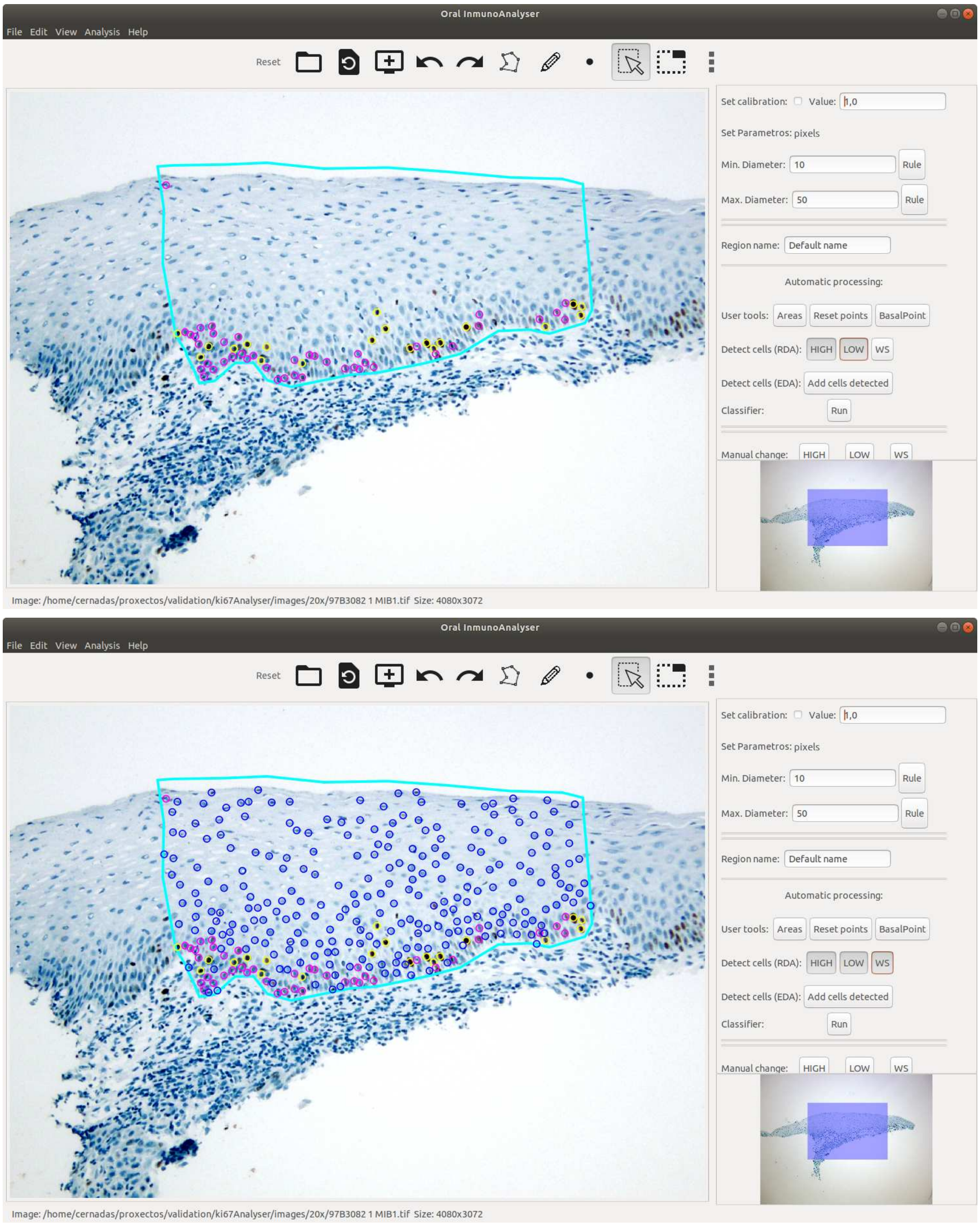

Figure S24. Image of the upper panel of figure S22 after the use the button **LOW** of RDA method (upper panel) and after the use of the three buttons of the RDA method (lower panel).

6. **Counts by subregions** (optional): OralImmunoAnalyser allows to count cells into the region of analysis by the following three subareas: basal, midle and superior (further details in section 9.2).
7. **Visualization of results** (optional): pressing the button **Show Table** of the lateral panel open a table at the bottom of window (see Figure S23) to show the following information: region name, number of cells in the region, percentage of cells high stained, percentage of cells low stained, percentage of cells without staining and the density of cells. The information shown in this table can be exported to a CSV file, as it has been described in the section 6.

9.1 Detect and classify cells

OralImmunoAnalyser encloses two differents algorithms to dectect the cells in the image, based on two different paradigms: 1) the method called RDA in the lateral panel (line eight); and 2) the method called EDA (line nine in the lateral panel). The RDA method searchs differences in some property among pixels in the image, while the EDA method tries to find the discontinuities between an object (in our case cell) and its neighborhood in some property. So, as both algorithms are based on different principles, it is possible that one algorithm works better on one type of images and the another on another type of images. It is important to emphasize that both methods are synchronized, which implies that the software checks if there is some overlapping among the detected cells by both methods (see below for examples).

Once the region of analysis was drawn and selected (as in the upper panel of figure S22), you can use the automatic detection of cells. Normally, the high stained cells are better detected by RDA algorithm (especially when the image is well-contrasted). The EDA methods detect better the non-stained cells (specially when image contrast is low), but it is rather sensitive to the image noise and, sometimes, provides an unwanted number of false positive. The more important is that both algorithms are quite fast and you can undo every automatic processing. So, the use of the four buttons of lines eight and nine in the lateral panel (buttons **HIGH**, **LOW**, **WS** and **Add detected cells**) can be used in any combination. All these buttons are toggle buttons, which mean that if the button is active, the detected cells are visualize on the image window and otherwise the detected cells are hidden (and you must active to visualize the cells again). The order in which the buttons are activated is also important due to the overlapping test, which is applied every time you modified the state of some button. The buttons **HIGH**, **LOW** and **WS** do not mean that the detected cells after clicking these buttons are the high stained, low stained and non-stained cells respectively (although the button **HIGH** detect the most stained cell, the **LOW** button a less stained cells and the **WS** the much less stained cells, but there are not absolute staining and they are only relative staining which depends on the image characteristics. Our purpose was to simulate the idea of the scale and to use different colours to draw the cells detected by each button in order to facilitate or visualize explicitly the cells detected clicking in the different buttons.

Some examples ilustrating the automatic detection of cells are shown in figures S22, S24 and S25. The lower panel of figure S22 shows the cells detected after clicking in the button **HIGH** (notice that the button is activated and the cells are shown as circles in yellow, which is the colour for category "high stained" in my working preferences). If you click again in the button **HIGH**, the button will desactivate and the detected cells by this button will be hidden. The figure S24 shows the cells added by clicking the button **LOW** in pink colour (upper panel) and the cells added by clicking the button **WS** in blue colour (lower panel). In both cases, the button is activated after clicking it. Although some cells can be detected cliking different buttons, notice that the cells never appear overlapped. The upper panel of figure S25 shows the detected cells added after clicking the button **Add detected cells** (method EDA) is red colour, which is the colour set to draw points in the working preferences. In this case, only one cell is added. If you have firstly applied this algorithm, the cells detected will be those shown in the lower panel of figure S25, but in the upper panel of figure S25, many of them were detected by RDA method before and then they are not added by the overlapping test. So, the order in which the four buttons are activated may provide a different set of cells automatically detected.

Figure S26 shows another example of operation of cell detection methods. In this case, the RDA method does not detect all cells, which can be detected by EDA method. After the cell detection process is concluded, the cells must be classified. For that, click the button **Run** after the label *Classifier* (see the section 11 for more details). Figure S27 shows the result of classification applied to the upper panel of

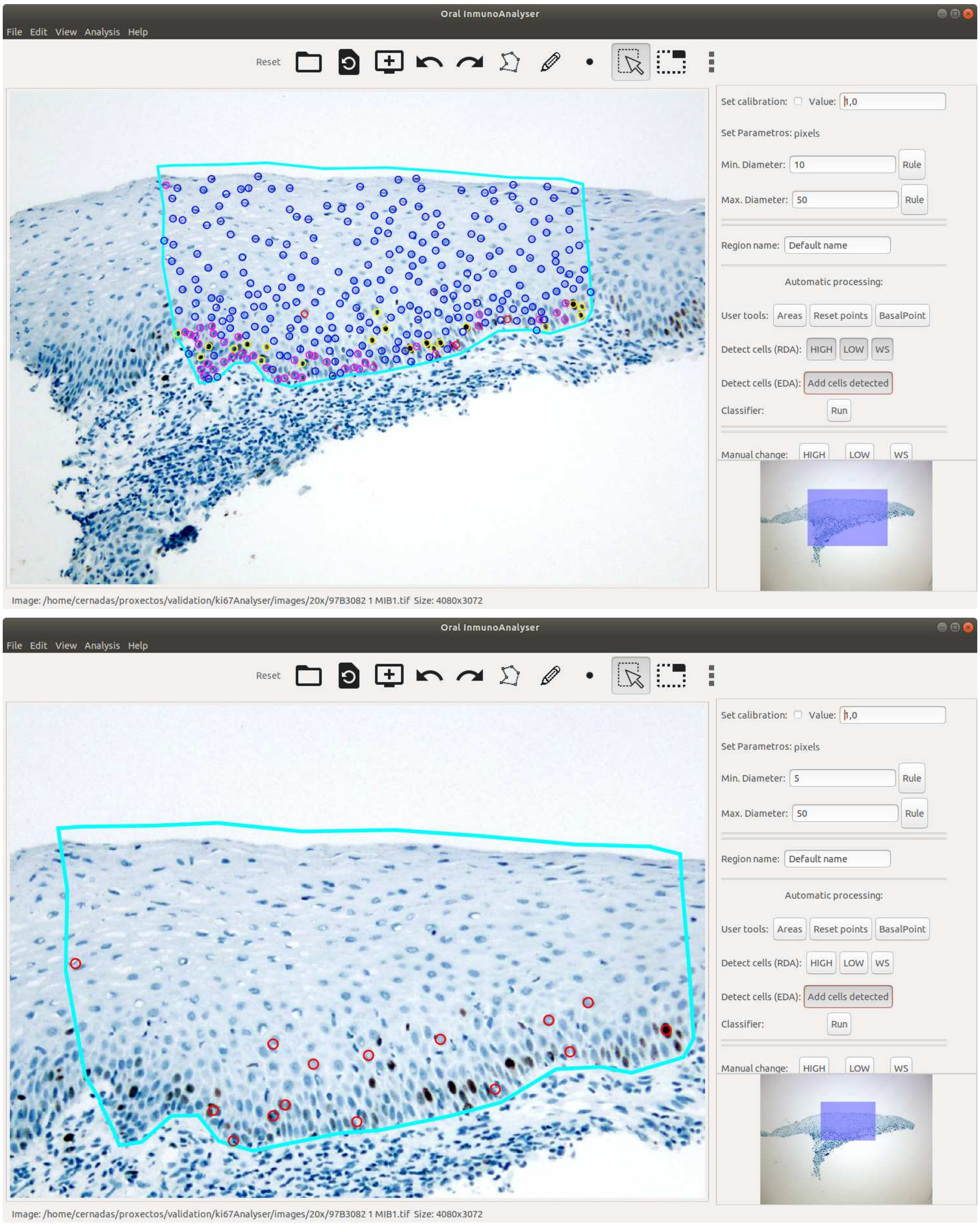

Figure S25. Image of the lower panel of figure S24 after adding the cells of EDA method (upper panel) and cells detected if you choose to apply the EDA method first (lower panel).

figure S25. The colours used were set in the working preferences (see section 5). In our preferences, the high stained cells are set to yellow, low stained cells are shown in pink and cells without staining are in blue colour. If the asigment of staining level is not suitable for the expert, OralImmunoAnalyser allows to change the category of the cells. To do that, the user must select the cells in two ways: 1) activate the select button in the toolbar and click near the point with the left button of the mouse (to select more than one cell, keep pressed the “Crtl” or “Control” key) and 2) use the penultimate button of toolbar to select the cells under the rectangle. Once the cell or cells are selected, click in some of the buttons after the

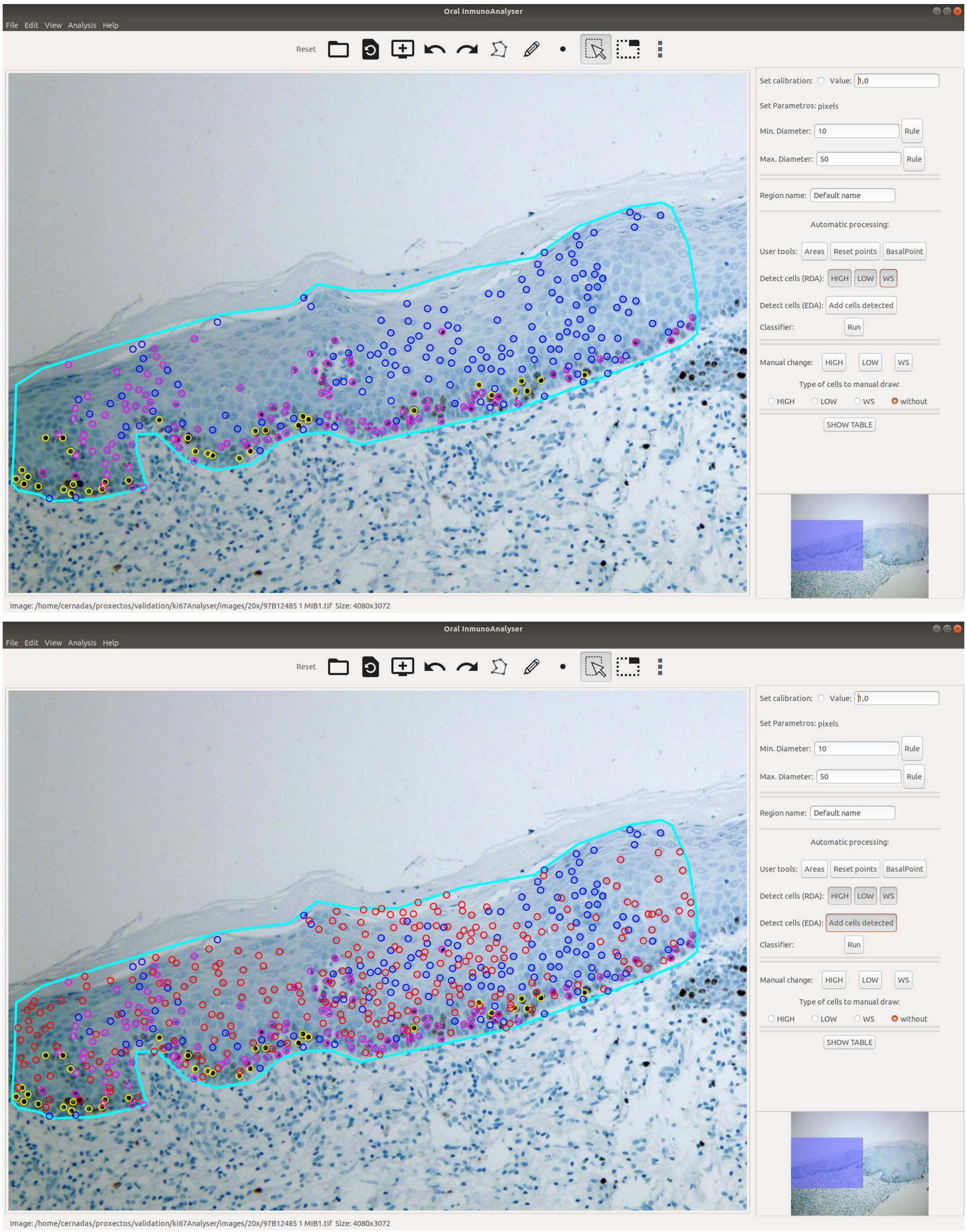

Figure S26. Example of the application of automatic detection of cells: (upper panel and points in yellow, pink and blue) after using RDA method and (lower panel and points in red) after adding the detected cells provided by EDA method.

label *Manual change* to change the category to the cells. Once the cells are selected, they can be removed clicking the botton “Supr”.

The faster way to add new cells of each category is following the next steps: 1) choose the category of cells to add clicking one of the radio buttons after the label *Type of cells for manual draw*; 2) activate the button to draw points (nineth button) in the toolbar; and 3) click points in the region of analysis (the points

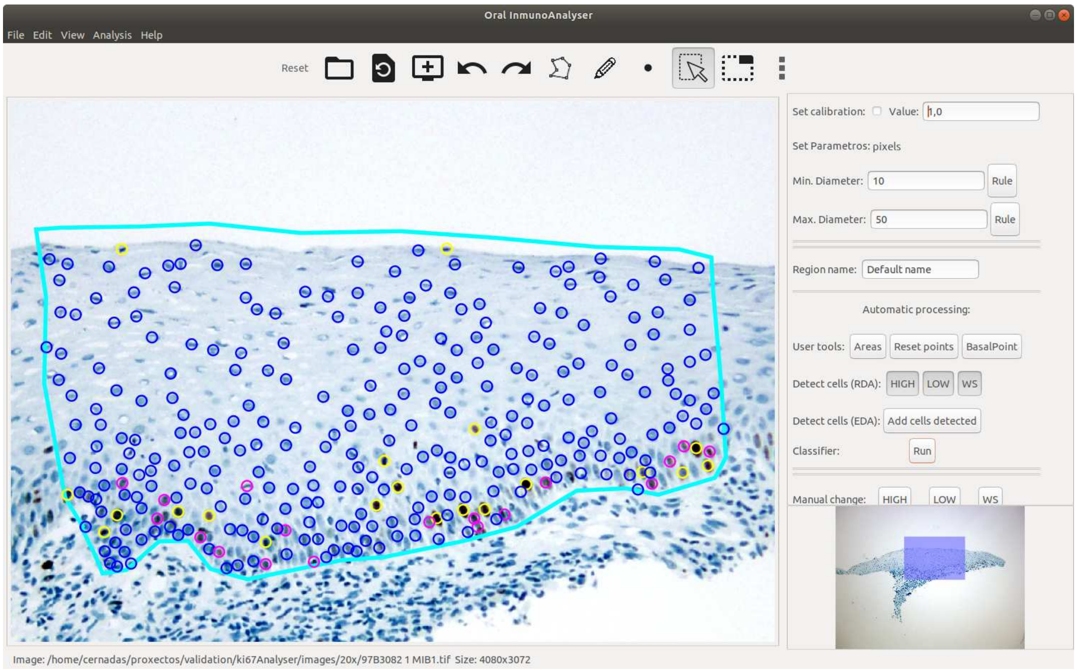

Figure S27. Image of the upper panel of figure S25 after using the automatic classification of cells, i. e. after clicking the button **Run** after the label *Classifier*.

added will visualize with the colour of the category chosen). It is recommended to choose the radio button **without** when you finish to add cells.

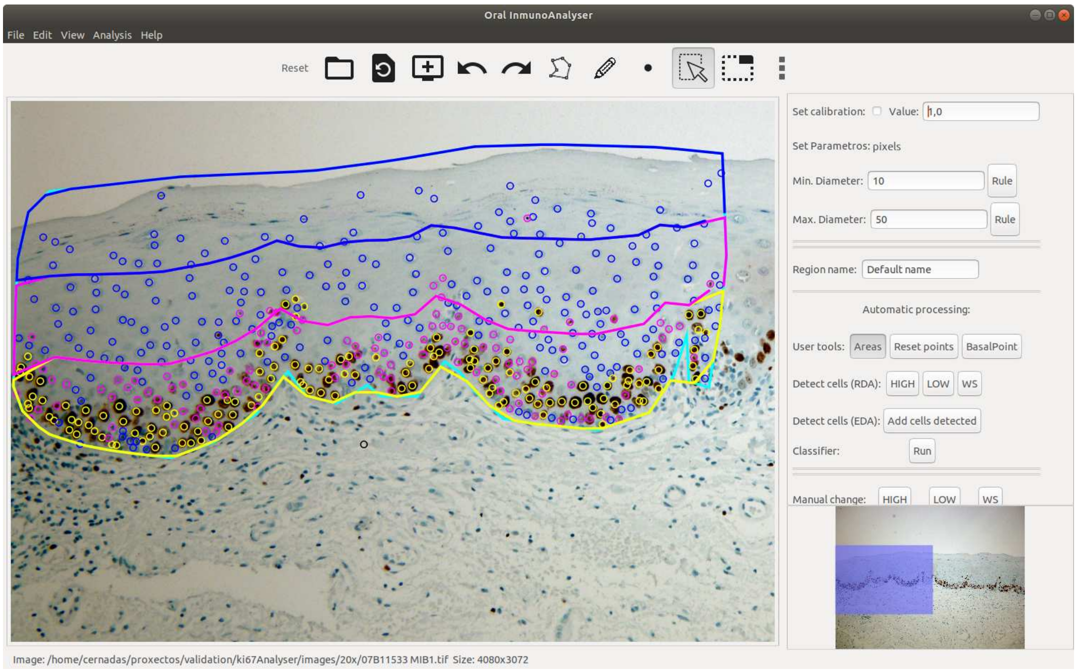

Figure S28. Show an example of the subareas basal, medial and superior calculated automatically in the region of analysis. The black point outside the region of analysis indicate to the computer the position of the basal subarea.

9.2 Counts by subareas

OralImmunoAnalyser allows to count the cells of each level of staining in the region of analysis, but also count the cells in the following subareas: basal, medial and superior. Each subarea covers approximatly the 33% of width of the region going from the basal to the superior part of the region. The subareas are automatically calculated by the computer following the next steps: 1) the user must provide to the computer the side of the region of analysis close to the basal area, marking a point in the image window outside the region of analysis; and 2) click the buton **Areas** after the label *User tools* to visualize the subareas calculated by the computer on the image window (the software used the categories colours to visualize each subarea). Although the subareas are visualized on the image window, their count is not shown in figure S23 due to computational issues, but this information is exported to CSV files. Figure S28 shows the subareas calculated by the computer (after marking the basal point) and figure S20 the count analysis exported to the CSV file. As mentioned, to indicate to the computer which side of the region of analysis is the basal, the user should do the following steps: 1) activate the button **BasalPoint** after the label *User tools* in the lateral panel; 2) while this button is active click a point with the left button of the mouse outside the region of analysis marking the basal side of the region (see the black circle in figure S28), when the point is marked the button **BasalPoint** will appear desactivated; and 3) click the button **Areas** after the label *User tools* to visualize the subareas calculated by the computer. The algorithm to calculate the subareas is not exhaustive and it could not work suitable if the region of analysis has folds or narrow valleys (as in the example shown in figure S21).

The button **Reset points** after the label *User tools* is used to clear all the cells into the region of analysis. To do that, you must select the region of analysis and then click this button to remove the cells in the selected region.

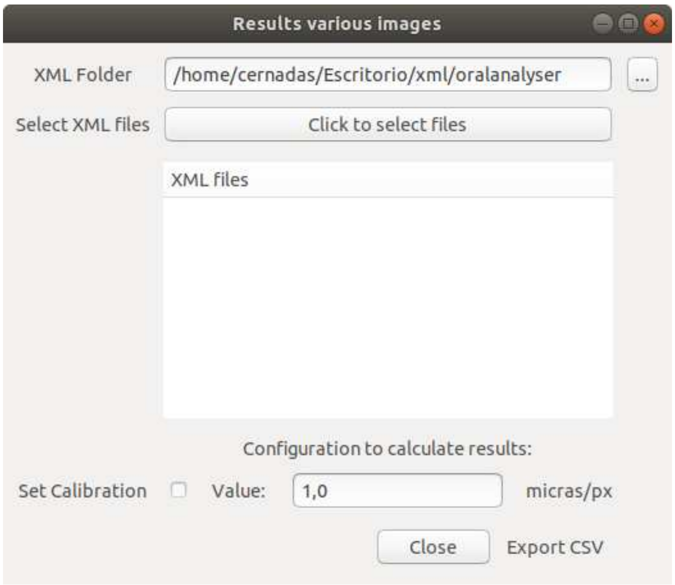

Figure S29. Window open when the submenu **XML File** of menu **Analysis** is chosen.

10 ANALYSIS MENU

The only item available is **XML File**, which opens the pop up window of figure S29. Many times the researchers want to acumulate the quantitative results of various images together. For this purpose, you must do the following steps: 1) process individually each image; 2) save the recognition results (region of analysis, basal point and the category of each cell in the region) in a XML file; and 3) run the submenu **XML File** of menu **Analysis** to calculate the jointly quantitative results.

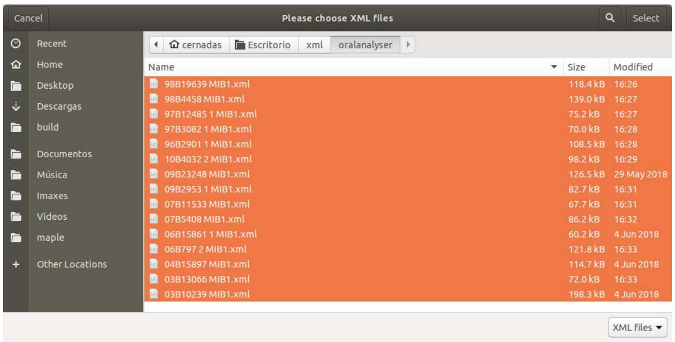

Figure S30. File chooser dialogue to choose the XML files included in the jointly analysis.

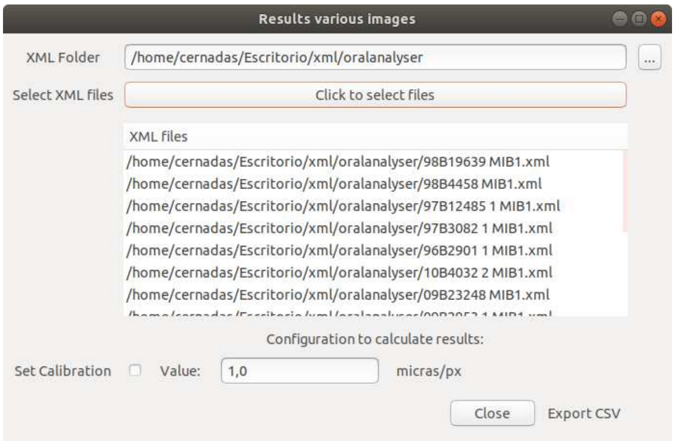

Figure S31. Window open when the submenu **XML File** of menu **Analysis** is chosen, after selecting the XML files used in the analysis.

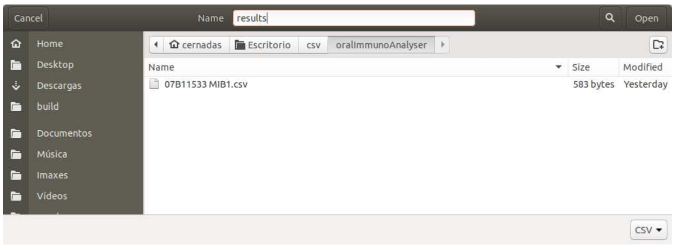

Figure S32. File chooser dialogue to provide the name of the CSV file.

The first line of window of figure S29, labelled as *XML Folder*, allows to choose the directory of the XML files (by default, it is the XML directory set in the preferences, but it can be changed clicking the three points button). The second line, labelled as *Select XML File*, have the button **Click to select files**. Clicking this button, the file chooser dialogue of figure S30 is open to choose the XML files used to compute the statistics results. As all file chooser dialogues, a file is selected if you click on the file name with the left button mouse. To choose consecutive files, you click the first file and, keeping the key **Alt** pressed, you click the last one. To choose various files, you click files names keeping the key **Ctrl** pressed. Once the XML files were chosen, click the **Select** button in the bottom of the window. The files selected will be appeared in the Figure S29, as it can be seen in the Figure S31. Then, the user can choose the following operations: 1) cancel this operation pressing the **Close** button in the bottom; or 2) press the **Export CSV** to store the joined statistical results in a CSV file. This last operation open a file chooser dialogue, which is shown in figure S32, providing the name `results` for the CSV file. This saving process of the `results.csv` file could require some seconds if there are many XML files to analyse. After saving one CSV file, the window of Figure S29 goes on open until the user click the **Close** button, in order to do other analysis. One example of the CSV file stored can be observed in the Figure S33. The information saved for each image are: 1) name of image; 2) name of the region of analysis and its number of cells; 3) area of region and density of cells (if the calibration is set it is appeared in micrometers and otherwise in pixels); 3) the global count of the image and the percentage of cells for each staining level; and 4) this count information for each subarea in the region. As it can be seen in figure S33, some rows are empty for the counts of the subareas (for example file `10B4032 2 MIB1.xml` in row 10). This means that the user has not provided the basal point and then has saved the overlays in the XML file. In this case, the subareas can not calculate the counts by subareas.

11 CLASSIFICATION MENU

As mentioned, OralImmunoAnalyser checks if the classifier was loaded when the user run OIA. If there is a classifier trained in the computer, the pop-up message of upper panel of figure S7 is shown, otherwise, it is shown the pop-up message of lower panel of figure S7 informing that the classifier must be trained before classifing cells.

The submenus of the Classification menu from the menu bar are:

1. **Classify**: assign a category (high, low or without stained) to each cell. This functionality can also do pressing the button **Run** after the label *Classifier* in the lateral panel (see the section 9.1). If there is no classifier loaded in OIA software, the pop-up message of figure S34 is shown. So, it is necessary to train the classifier before using it.
2. **Train classifier**: train the classifier, which is explaining below.

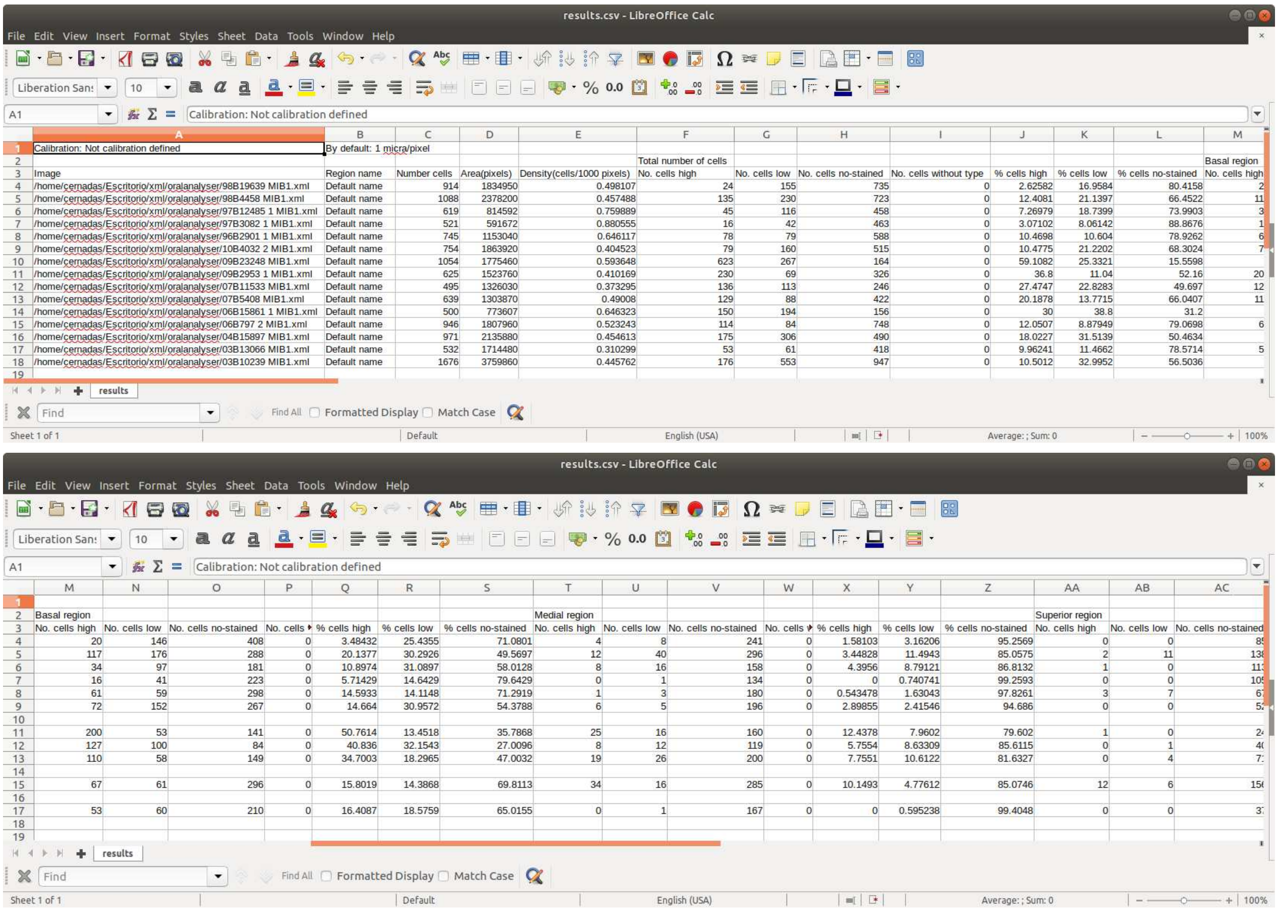

Figure S33. An example of CSV file of the analysis of various XML files imported in LibreOffice Calc. The lower screenshot is the continuation of the upper screenshot.

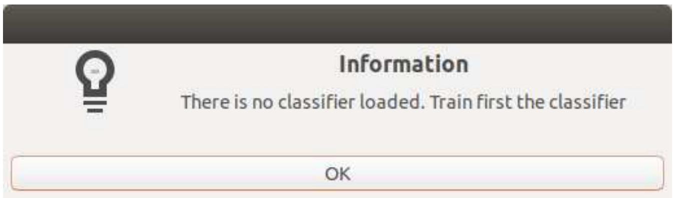

Figure S34. Pop-up window to inform that there is no classifier loaded.

The establishment of the stained level of the cell (into category high stained, low stained and without staining) can be manually set in every time using the GUI (as it was described in section 9.1). But, OralImmunoAnalyser implement the automatic classification of cells. Before applying the classification operation (using the submenu **Classify** or click the button **Run** in the lateral panel after the label “Classifier”), the classifier need to be trained. The classifier is a supervised machine learning tecnique, which need to learn a function to predict the category of the cell, which have not been seen before by the classifier. To learn this function it is necessary to provide to the classifier a set of cells for which the category is known in a process called training. The image characteristics used to discriminate different categories of cells are color features extracted from the image in the position of the cells. So, you must manually analyse a limited number of cells and save the annotated cells overlapped to the image in their

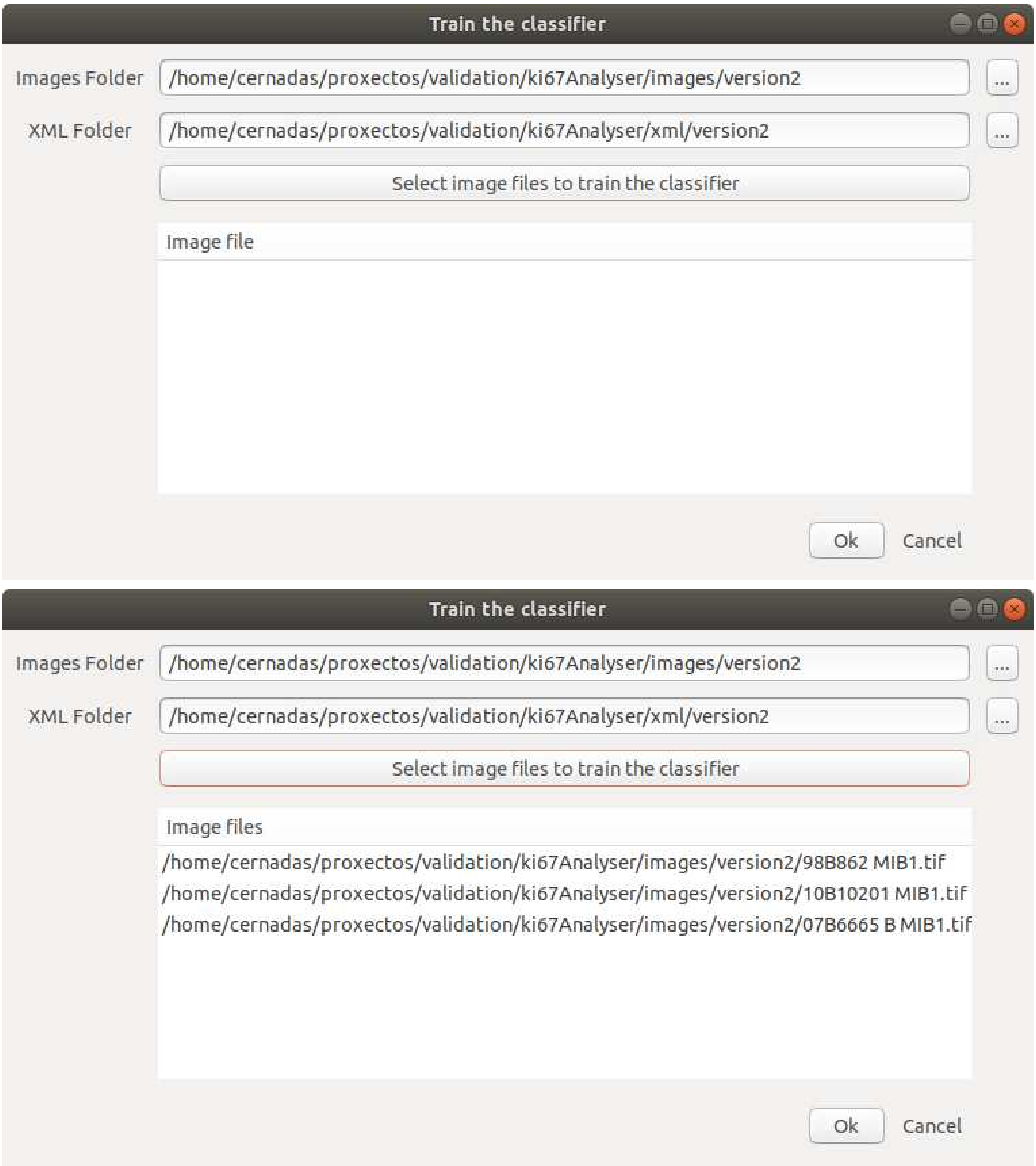

Figure S35. Window open to train the classifier: before (upper panel) selection the image files and after (lower) selecting the image files to train the classifier.

corresponding XML files (using the default name, which is the image name with the extension `.xml`) in order to train the classifier. Afterwards, to train the classifier do the following steps:

1. Select the submenu **Train classifier** in menu **Classification**, which opens the window of the upper panel of figure S35. The first line allows to select the image folder, which is, by default, the image folder specified in the working preferences (see section 5). The second line allows to select the XML

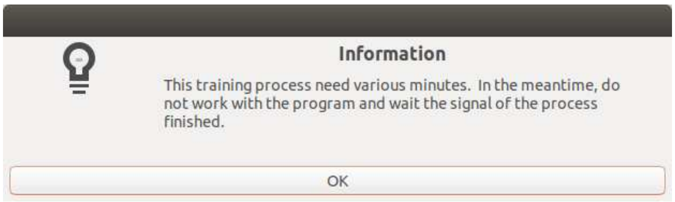

Figure S36. Pop-up window to inform that the training process need various minuted to finish.

folder, which is also the folder specified in the preferences by default. As we mentioned in section 5 (see the figure S10), clicking in the button with three points, you can change the image and XML folders.

2. Click the button **Select images files to train the classifier**, which opens a file choose dialogue as in the figure S30 to select the image files used to train the classifier. The lower panel of figure S35 shows the figure of upper panel after the selection of the image files.
3. Click the button **Cancel** to cancel the training process or the button **Ok** to train the classifier. After clicking the button **Ok**, the pop-up window of figure S36 informs that the training process need various minutes depends on the number of images used in the training, the number of cells and the complexity of the problem to learn for the classifier). While the OralImmunoAnalyser is trainig its classifier, you can not use it. When the training process finished, the pop-up window of upper panel of figure S7 will be shown.
4. Once the classifier was trained, OIA assign a category to the cell clicking the button **Run** after the label *Classifier* in the lateral panel or going to submenu **Classify** in menu **Classification**. Obviously, you can only apply the classification process if there is a region of analysis selected with cells recognized on the image window.

12 HELP MENU

The **Help** menu is within the menu bar with the submenus **User Manual** and **About Us**. The submenu **About Us** pop-ups a window with a decription of OralImmunoAnalyser and informations about thanks and licence (see Figure S37). The submenu **User Manual** does not do anything. The help to the users are provided in the current user guide. As well, if you put the mouse pointer on some widget (button, entry, label, etc) of the GUI, a pop-up message are shown with information about the functionality of that widget.

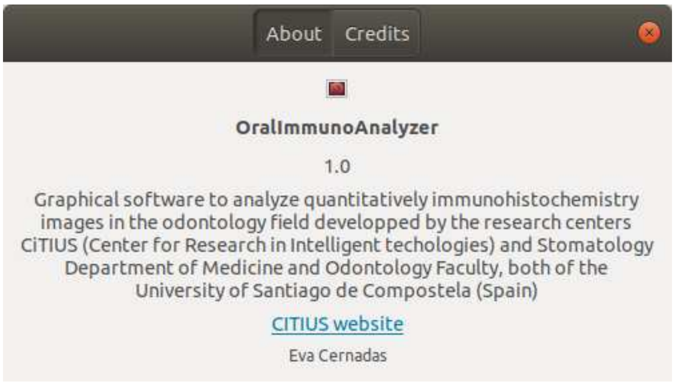

Figure S37. Pop up dialogue to inform about help.
